# α-hederin Targets USP5 to Inhibit Colorectal Tumorigenesis by Disrupting STAT3 Deubiquitination

**DOI:** 10.7150/ijbs.119868

**Published:** 2025-10-20

**Authors:** Hui Feng, Qijuan Wang, Liu Li, Lihuiping Tao, Shuhong Zeng, Ziwen Li, Minmin Fan, Chengtao Yu, Dongdong Sun, Weixing Shen, Haibo Cheng

**Affiliations:** 1 The First Clinical Medical College, Nanjing University of Chinese Medicine, Nanjing 210023, China.; 2 Zhenjiang Hospital of Chinese Traditional and Western Medicine, Zhenjiang 212000, China.; 3 Jiangsu Collaborative Innovation Center of Traditional Chinese Medicine in Prevention and Treatment of Tumor, Nanjing 210023, China.; 4 School of Integrated Chinese and Western Medicine, Nanjing University of Chinese Medicine, Nanjing 210023, China.; 5 Department of Oncology, Affiliated Hospital of Nanjing University of Chinese Medicine, Nanjing 210023, China.

**Keywords:** colorectal cancer, STAT3, USP5, deubiquitination, α-hederin

## Abstract

α-hederin is a natural compound that is used to treat colorectal cancer (CRC). However, the precise anti-CRC mechanism needs to be explored further, and its direct targets have not yet been reported. In the present study, for the first time, we revealed that α-hederin directly targeted ubiquitin specific peptidase 5 (USP5), decreased its expression, weakened its interaction with signal transducer and activator of transcription 3 (STAT3), and disrupted STAT3 deubiquitination, thereby inhibiting colorectal tumorigenesis. This is particularly significant because STAT3 is a key mediator of inflammation and tumorigenesis, and targeting STAT3 deubiquitination represents a promising pathway for combating CRC; however, its deubiquitination mechanism in CRC remains unclear. USP5, a deubiquitinating enzyme (DUB) involved in inflammatory responses that is highly expressed in primary CRC tissues and promotes tumorigenesis by stabilizing tumor proteins, was identified in our study as a novel DUB of STAT3. In addition, we showed that USP5 serves as an oncogene in CRC by deubiquitinating STAT3, which contributes to CRC progression. Overall, our study provided evidence that α-hederin exhibits significant potential in suppressing colorectal tumorigenesis by disrupting USP5-mediated STAT3 deubiquitination.

## Introduction

Colorectal cancer (CRC) is one of the most serious malignant tumors worldwide, with the third highest incidence and second highest mortality rates among all malignant tumors 1. Chronic inflammation is a risk factor for a high incidence of CRC, which damages the intestinal mucosa, causing abnormal proliferation and accelerated transit of intestinal epithelial cells (IECs), ultimately leading to colorectal tumorigenesis 2. Reducing the incidence of chronic inflammation and inhibiting the activation of the inflammation-cancer transition signaling pathway are hotspots for CRC prevention and treatment.

Therefore, the identification and development of drugs that halt the progression of CRC are critical for reducing the incidence of CRC. However, the efficacy of many clinical drugs is not impressive, and many promising drugs have failed to reach the clinical trial stage because of resistance mechanisms and adverse effects 3. Natural products play an important role in oncology because of their efficacy and safety. Among them, α-hederin is a monodesmosidic triterpenoid saponin derived from *Fructus Akebia* that has been used in CRC treatment 4-11. In our previous study, we have confirmed that α-hederin has the effect of inhibiting the malignant transformation of IECs 12, 13. However, the potential role of α-hederin in colitis carcinoma transformation progression has rarely been described, and the precise anti-CRC mechanism and direct targets of α-hederin in CRC have not been ascertained.

Notably, signal transducer and activator of transcription 3 (STAT3) is a key mediator of inflammation and tumorigenesis and is required for the survival and proliferation of intestinal epithelial cells (IECs) that initiate tumors 14, 15. The inhibition of the STAT3 pathway can significantly reduce the incidence of colitis-associated CRC. STAT3 is a transcription factor that enters the nucleus and regulates the expression of various genes, thereby affecting physiological processes such as cell growth, inflammatory response, and immunomodulation 16. As an oncogene, STAT3 is overexpressed in tumor biopsies from patients with CRC 14 and is activated during colorectal tumorigenesis 15. Its activation is typically constitutive and requires phosphorylation of a key tyrosine residue (Tyr705) 17. Therefore, STAT3 has emerged as an attractive molecular target, and the identification or development of compounds that inhibit STAT3 signaling is one of the most popular strategies for CRC prevention and treatment. Notably, growing evidence indicates that targeting STAT3 ubiquitination is an alternative pathway for combating human malignancies 18, 19, and plays an important role in regulating STAT3 stability. However, the mechanisms underlying STAT3 ubiquitination in CRC remain unclear. As a post-translational modification (PTM), ubiquitination is a reversible process catalyzed by E3 ubiquitin ligases but counter-regulated by deubiquitinating enzymes (DUBs) 20.

DUBs remove the ubiquitin chain, reverse the ubiquitination, and increase the stability of substrate proteins. Importantly, a growing number of studies have shown that aberrant DUBs function is associated with a wide range of human diseases, including cancer 21, 22, opening the possibility of DUBs as attractive drug targets for clinical translation 23, 24. Ubiquitin specific peptidase 5 (USP5) is an important member of the ubiquitin-specific protease (USP) family, that is the largest family of DUBs 22, which is involved in the ubiquitin pathway that promotes tumorigenesis by deubiquitinating and stabilizing tumor proteins 25 and is involved in inflammatory responses 26. Previous study has reported that USP5 was highly expressed in primary CRC tissues of patients, and correlated with disease stage and overall survival 27. It further revealed that USP5 promoted tumor cells growth and enhanced their resistance to the chemotherapeutic drug doxorubicin by deubiquitinating Tu translation elongation factor (TUFM). However, its mechanism of action in CRC is yet to be thoroughly investigated. Therefore, identifying the substrates of USP5 in CRC is critical for exploring its mechanism of action and developing novel therapeutic strategies. To date, ubiquitin specific peptidase 28 (USP28), which is homologous to USP5, is the only known DUB of STAT3 and has been studied in non-small-cell lung cancer (NSCLC) 19. Therefore, the mechanisms of USP5 deubiquitination and STAT3 ubiquitination in CRC remain a gap, and making progress in these areas will contribute to the prevention and treatment of CRC, potentially shedding light on the mechanism of action of α-hederin.

Here, we aimed to identify the direct targets of α-hederin and to explore and reveal the effect and mechanism of α-hederin on CRC progression in azoxymethane/dextran sodium sulfate (AOM/DSS) model.

## Materials and Methods

### Reagents and antibodies

Azoxymethane (AOM) (A5486) was supplied by Sigma-Aldrich (St. Louis, MO, USA), and dextran sulfate sodium (DSS) (36-50 kDa) was obtained from MP Biomedicals (Morgan Irvine, CA, USA, MP0216011080). α-hederin was purchased from Chengdu Herbpurify CO., Ltd. (purity > 98%) and dissolved in dimethyl sulfoxide (DMSO) (Solarbio, Beijing, China, D8371) to indicated concentrations. MG132 (ab141003) was purchased from Abcam (Cambridge, UK). Cycloheximide (HY-12320), Pronase E (HY-114158), Stattic (HY-13818), and Colivelin (HY-P1061) were obtained from MedChemExpress (Monmouth Junction, NJ, USA). The 3-(4,5-dimethylthiazol-2-yl)-2,5 diphenyl tetrazolium bromide (MTT) (M2128) and lipopolysaccharide (LPS) (L5293) were purchased from Sigma-Aldrich (St. Louis, MO, USA). D-biotin was supplied by Thermo Scientific (Waltham, MA, USA, B1595). N-Boc-2,2'-(ethylenedioxy) diethylamine (N854951), NHydroxybenzotrizole (HOBT) (H810970), 1-Ethyl-3-(3-dimethylaminopropyl) carbodiimide hydrochloride (EDCI) (N835594), N,N-Diisopropylethylamine (DIPEA) (N766991), N,N-dimethylformamide (DMF) (N807505), and dichloromethane (DCM) (D824411) were purchased from Macklin Biochemical Technology (Shanghai, China). Hydrogen chloride 1,4 dioxane solution was supplied by Energy Chemical (Shanghai, China; W810049). Antibodies against c-Myc (YT0991) was purchased from Immunoway (Plano, TX, USA), Ki67 (TW0001), IL-6 (TD6087), β-Tubulin (M20005), STAT3 (T55292F), p-STAT3 (Tyr705) (T56566F) and NF-κB (T55034) were obtained from Abmart (Shanghai, China), β-catenin (Santacruz, sc-7963), PCNA (ab18197), p62 (ab207305), p38 (ab170099), IL-10 (ab9969), Ubiquitin (ab134953), Cyclin D1 (ab134175), CDK1 (ab265590), Bcl-2 (ab182858), Alexa Fluor^®^ 594 labeled Goat anti-rabbit IgG (H+L) (ab150080) and Alexa Fluor^®^ 488 labeled Goat anti-mouse IgG (H+L) (ab150113) were supplied by Abcam (Cambridge, UK), TNF-α (60291-1-Ig), IL-1β (26048-1-AP), ZO-1 (21773-1-AP), Occludin (66378-1-Ig) and USP5 (10473-1-AP) were obtained from Proteintech (Wuhan, China), Beclin 1 (CY5092) was purchased from Abways (Shanghai, China), p-p38 (Thr180/Tyr182) (4511T), p-NF-κB (Ser536) (13346S), Bax (2772T), anti-mouse IgG (7076P2) and anti-rabbit IgG (7074P2) were provided by CST (Danvers, MA, USA).

### Cell culture

Five human CRC cell lines (Lovo, Caco-2, RKO, HCT8, and HCT116) and three normal human IECs (FHC, FHs 74 Int, and NCM460) were obtained from the American Type Culture Collection (ATCC, Manassas, VA, USA). The cells were cultured respectively in Ham's F-12K, Dulbecco's modified Eagle medium (DMEM), DMEM, DMEM, McCoy's 5A, DMEM, DMEM, and RPMI-1640 medium containing 10% fetal bovine serum and 1% penicillin-streptomycin at 37°C in humidified air containing 5% CO_2_.

### Cell viability assay

Cells (NCM460, FHC, and HCT116) were seeded at a density of 8 × 10^3^ cells /well in a 96-well plate and treated with 0, 5, 10, 20, 40, 80 μmol·L^-1^ of α-hederin for 24 h and 48 h, respectively. Subsequently, MTT (5 mg·mL^-1^) was added, and the cells were incubated for 3 h. The cell supernatant was removed and 200 μL of DMSO was added to dissolve the resulting formazan precipitate, and the absorbance at 490 nm was measured using a microplate reader (TECAN SPARK 10M, Männedorf, Switzerland).

### LPS induced enteritis model of intestinal epithelial cell NCM460* in vitro*

NCM460 cells were stimulated with LPS (2 μg·mL^-1^) for 24 h. In α-hederin-treated groups, cells were stimulated for 24 h with different concentrations.

### Protein isolation and western blotting

The cells and colon tissues were lysed on ice with appropriate volume of cell lysis buffer (Beyotime, Shanghai, China, P0013J) and RIPA lysis buffer (Beyotime, Shanghai, China, P0013B), respectively. Both buffers contained proteases (Roche, Basel, Switzerland, USA, 11836153001) and phosphatase inhibitors (Roche, Basel, Switzerland, USA, 4906845001). Protein concentrations were determined by a bicinchoninic acid (BCA) protein assay kit (Thermo Scientific, Waltham, MA, USA, 23225). Total proteins were separated using sodium dodecyl sulfate-polyacrylamide gel electrophoresis (SDS-PAGE) and transferred onto polyvinylidene difluoride (PVDF) membranes. Membranes were blocked in phosphate-buffered saline with Tween 20 (PBST) containing 5% skim milk at room temperature (RT) for 2 h, followed by incubation with primary antibodies overnight at 4 °C. The membranes were then incubated with secondary antibodies at RT for 1 h. After incubation with ECL substrate (Biosharp, Beijing, China, BL520B), the membranes were visualized using a Chemi Doc XRS+ imaging system (Bio-Rad, Hercules, CA, USA).

### RNA isolation and quantitative real-time polymerase chain reaction‌ (qPCR)

Cell and colon tissue samples were isolated using TRIzol reagent (Invitrogen, Carlsbad, CA, USA, 15596018). RNA was reverse-transcribed to complementary DNA (cDNA) using the Evo M-MLV RT Mix Kit with gDNA Clean for qPCR (ACCURATE BIOTECHNOLOGY, CO., Ltd., Changsha, Hunan, China, AG11728). Then qPCR was performed using the SYBR Green Premix Pro Taq HS qPCR Kit (ACCURATE BIOTECHNOLOGY, CO., Ltd., Changsha, Hunan, China, AG11701) in a StepOne Plus real-time PCR system (Applied Biosystems, Foster, CA, USA). The 2^-ΔΔCt^ method was used to evaluate relative gene expression, and β-actin was used as an internal control. The primers used are listed in **Supplementary Table S1**.

### Luciferase assay

A total of 5 × 10^4^ NCM460 cells/well were plated into 12-well plates, and transfected with pSTAT3-TA-Luc (Beyotime, Shanghai, China, D4404) or pNF-κB-Luc (Beyotime, Shanghai, China, D2206) through Lipofectamine 8000 (Beyotime, Shanghai, China, C0533), according to the manufacturer's instruction. This was performed when cells reached 70% confluence, followed by incubation with LPS (2 μg·mL^-1^) and α-hederin (2.5, 5, or 10 μmol·L^-1^) for 24 h. Next, luminescence intensities of cell lysates were measured using the Firefly Luciferase Reporter Gene Assay Kit (Beyotime, Shanghai, China, RG005), according to the manufacturer's protocol.

### Animal experiment

Six-week-old female specific pathogen-free (SPF) grade C57BL/6 mice were purchased from WeiTong LiHua Laboratory Animal Technology Co., Ltd. (Beijing, China). Animal welfare and experimental procedures were performed in strict accordance with the Guide for the Care and Use of Laboratory Animals (National Institutes of Health, USA) and were approved by the Animal Care and Use Committee of Nanjing University of Chinese Medicine (Approval Number: 202207A077). All efforts were made to minimize animal suffering and to reduce the number of animals used. Mice were adapted to the laboratory environment of the Experimental Animal Center of Nanjing University of Chinese Medicine for one week before grouping based on similar body weights for the experiment, with free access to water and food before the commencement of the animal experiment. To induce CRC, mice were injected intraperitoneally (i.p.) once with 10 mg·kg^-1^ AOM. Seven days after AOM injection, the mice were subjected to three-cycle of one week 2.5% DSS solution administration followed by normal drinking water for two weeks 28. Mice were randomly divided into four groups (n = 5): control group, model (AOM/DSS) group, AOM/DSS+α-hederin low dose (α-hederin L) group, AOM/DSS+α-hederin high dose (α-hederin H) group. The α-hederin L group was injected (i.p.) with 1 mg·kg^-1^ α-hederin and the α-hederin H group was injected (i.p.) with 4 mg·kg^-1^ α-hederin every other day. Mice in AOM/DSS+α-hederin groups were given α-hederin treatment starting the day after intraperitoneal injection of AOM, throughout the entire duration of the experiment-16 weeks prior to being subjected to DSS stimulation. Body weight was measured every two days during the treatment period. At the end of the 16th week, the mice were euthanized. The colon was harvested, rinsed with phosphate-buffered saline (PBS), incised longitudinally along the primary axis, and the number and size (long diameter of polyps) of tumors formed in the colon were analyzed. The liver, spleen, and kidneys were weighed, and blood was harvested for a follow-up study.

### Hematoxylin and Eosin (H&E) staining

The colonic tissue from each mouse was fixed with 4% paraformaldehyde (PFA) and embedded in paraffin. Colon slices (4 μm) were stained with H&E and photographed by a fully automated quantitative pathology imaging system (Vectra 3.0, PerkinElmer, USA), and graded using the method described by Hozumi 29.

### Immunofluorescence (IF) analysis

Cells were fixed by 4% PFA, and permeabilized with Saponin (Beyotime, Shanghai, China, P0095) and blocked with 5% bovine serum albumin (BSA), whereas the paraffin-embedded sections of the colon were hydrated by serial dewaxing, followed by antigen retrieval using citric acid antigen repair solution (pH 6.0) (Solarbio, Bejing, China, G1202), and then blocked with 3% goat serum (Beyotime, Shanghai, China, C0265) at RT for 1h. Subsequently, incubation at 4 °C overnight with primary antibodies was performed and incubated with Alexa Fluor-labeled secondary antibodies at RT for 2 h. Finally, the nuclei were stained with antifade mounting medium with 2-(4-Amidinophenyl)-6-indolecarbamidine dihydrochloride (DAPI) (Beyotime, Shanghai, China, P0131) for 10 min, and images were captured using an inverted fluorescent microscope (THUNDER Imaging Systems CN, Leica, Wetzlar, Germany) or a confocal laser-scanning microscope (Leica, Wetzlar, Germany).

### Immunohistochemistry (IHC) analysis

Paraffin slices of the colon tissue were deparaffinized in xylene and rehydrated using graded alcohol. Next, the colon slices were boiled for 10 min in citric acid antigen repair solution (pH 6.0) (Solarbio, Bejing, China, G1202). After blocking endogenous peroxidase with 3% hydrogen peroxide for 10 min, the slices were blocked with 3% goat serum (Beyotime, Shanghai, China, C0265) at RT for 1h and then incubated with the indicated primary antibodies overnight at 4 °C and incubated with secondary antibodies at RT for 1 h. Finally, the sections were treated with 3,3-N-DiaminobenzidineTertrahydrochloride (DAB) substrate-chromogenic solution (Beyotime, Shanghai, China, P0202) for 10 min, and stained with hematoxylin staining solution (Beyotime, Shanghai, China, C0107) for nuclear staining. Images were captured using a fully automated quantitative pathology imaging system (Vectra 3.0, PerkinElmer, USA).

### Enzyme-linked immunosorbent assay (ELISA)

The content of interleukin-6 (IL-6) (EK206), interleukin-1 beta (IL-1β) (EK201B), tumor necrosis factor-alpha (TNF-α) (EK282), and interleukin-10 (EK210) in serum was detected by indicated ELISA kits (Lianke, Hangzhou, China) according to the manufacturer's instructions. The content of IL-6 (AF0049), IL-1β (AF0179), and TNF-α (AF0121) in cell culture supernates was detected by indicated ELISA kits (Aifang Biotechnology, Changsha, Hunan, China) according to the manufacturer's instructions.

### Measurement of oxidative stress

The frozen sections (8 μm) were rewarmed, circled with a histochemical pen, and incubated with well-diluted dihydroethidium (DHE) (Beyotime, Shanghai, China, S0063) in PBS (PBS: DHE = 200:1) for 30 min at 37 °C under light-protected conditions. Next, the sections were washed in PBS on a decolorizing shaker thrice for 5 min each time. The sections were shaken slightly, and the nuclei were stained with an antifade mounting medium containing DAPI for 10 min at RT in the dark. Finally, the sections were observed using an inverted fluorescence microscope (Nikon Eclipse Ti, Nikon, Tokyo, Japan), and images were captured.

### Co-immunoprecipitation (Co-IP)

Proteins were extracted using a lysis buffer supplemented with protease and phosphatase inhibitors. Protein concentrations were measured using the BCA protein assay kit. Each sample was incubated with the indicated primary antibodies and protein A + G magnetic beads (Beyotime, Shanghai, China, P2179) overnight at 4°C under rotary agitation. Proteins that were not immobilized on the beads were removed by washing thrice with cold lysis buffer. Next, the precipitates were boiled for 10 min in SDS-loading buffer and detected using western blotting.

### Silver staining and liquid chromatography-mass spectrometry/mass spectrometry (LC-MS/MS) analysis

Total protein was extracted from HCT116 cells, and immunoprecipitation (IP) was performed using an anti-USP5 antibody or anti-IgG antibody and protein A + G magnetic beads, as described above. The immunoprecipitates were collected and visualized by 10% SDS-PAGE, followed by silver staining with a Fast Silver Stain Kit (Beyotime, Shanghai, China, P0017S). The anti-USP5 immunoprecipitation-specific protein bands were cut from the gel for LC-MS/MS analysis, which was conducted by Luming Biotechnology CO., Ltd. (Shanghai, China).

### Synthesis of biotin-conjugated α-hederin (Bio-α-hed)

The coupling reaction was performed using 4.1 mmol D-biotin, 3.4 mmol N-Boc-2,2'-(ethylenedioxy) diethylamine, 6.7 mmol HOBT, 6.7 mmol EDCI in DIPEA and DMF solution for reaction at RT for 12 h. The reaction was quenched with saturated ammonium chloride solution, diluted with water, extracted by DCM, the organic phase was washed with saturated ammonium chloride and saturated sodium chloride, dried with anhydrous sodium sulfate, and concentrated by filtration and silica gel column chromatography to obtain a white solid, N-biotinyl-N-Boc-2,2'-(ethylenedioxy) diethylamine, which was dissolved in hydrogen chloride-dioxane solution and stirred at RT for 12 h. After completion of the reaction, the solution was evaporated and dried under vacuum followed by the addition of 0.42 mmol α-hederin, 0.84 mmol EDCI, 0.84 mmol HOBT in DIPEA, DMF, and DCM solution for reaction at RT for 24 h. Bio-α-hederin was purified using high-performance liquid chromatography and analyzed by mass spectrometry.

### Proteome microarray assay

HuProt^TM^ V4.0 21K human proteome microarrays (Tibikang Biotechnology, Guangzhou, China) were used to identify α-hederin-interacting proteins. Microarrays were blocked with 3% BSA in PBS at RT for 1 h, and then incubated with Bio-α-hederin or D-biotin diluted to a final concentration of 10 μmol·L^-1^ with 1% BSA in PBST at RT for 1 h. After washing in PBST, the chips were co-incubated with Cy3-Streptavidin (Cy3-SA) (Sigma-Aldrich, St. Louis, MO, USA, S6402, 1:1000) at RT for 1h away from light. After washing in PBST, chips were dryed with SlideWasher (CapitalBio Technology, Beijing, China), and scanned by Luxscan^TM^ 10Κ-A (CapitalBio Technology, Beijing, China) to interpret signals and convert them into data. Signal-to-noise ratio (SNR) was defined as the mean ratio of foreground to background values for two replicate spots for each protein. For any protein, the normalized SNR (NOR-SNR) value of Bio-α-hederin was calculated, and the cutoff-value ≥ 1.5 was set to screen potential positive proteins for α-hederin. To identify specifically bound target proteins, for screened positive proteins, the fold change of the difference between the NOR-SNR value of Bio-α-hederin and D-biotin was calculated, and positive proteins with fold change (FC) ≥ 1.2 were defined as positive binding proteins of α-hederin. Data were analyzed using GenePix Pro 6.0.

### Molecular docking

The 2D structure of natural compound α-hederin was obtained from PubChem (PubChem CID: 73296). The 3D crystal structure of USP5 was downloaded from the Protein Data Bank (PDB ID: 7MS7), and the 3D crystal structure of STAT3 was downloaded from the Protein Data Bank (PDB ID: 6NJS). USP5 protein was assigned as the receptor, and α-hederin or STAT3 was assigned as the ligand. Compound-protein interaction prediction was performed using the ZDOCK server (https://zdock.umassmed.edu/), and docking was performed in AutoDock 2 with coarse docking using a simulated annealing algorithm and subsequent refinement using a genetic algorithm. Protein-protein interaction prediction was performed using the HDOCK web service (http://hdock.phys.hust.edu.cn/), and the binding results were visualized using PyMol software.

### Cellular thermal shift assay (CETSA)

HCT116 cells were cultured in 100-mm dishes and treated with DMSO or without α-hederin (10 μmol·L^-1^) for 4 h followed by digestion with trypsin, resuspended with PBS supplemented with protease and phosphatase inhibitors, and evenly distributed into 10 PCR tubes, and heated in a PCR machine (Bio-Rad, Hercules, CA, USA) for 5 min at 46 °C, 49 °C, 52 °C, 55 °C, 58 °C, 61 °C, 64 °C, 67 °C, 70 °C, and 73 °C. Subsequently, the cells were subjected to 3 cycles of freeze-thawing with liquid nitrogen to obtain cell lysates, followed by centrifugation at 12,000 rpm for 10 min. Finally, the samples were analyzed by immunoblotting (IB) with USP5 antibody.

### Drug affinity responsive target stability (DARTS)

HCT116 cells were lysed in RIPA buffer supplemented with protease and phosphatase inhibitors. The lysates were equally aliquoted into five Eppendorf tubes followed by incubation with DMSO and α-hederin (5, 10, 20 μmol·L^-1^) at RT for 1 h with gentle shaking, and subjected to pronase E (25 µg·mL^-1^) for proteolysis at RT for 30 min. Subsequently, the samples were mixed with SDS-loading buffer, heated at 95 °C for 10 min, and analyzed using IB assay to detect USP5 expression.

### Streptavidin pull-down assay

The cells and colon tissues were suspended in lysis buffer containing protease and phosphatase inhibitors. The supernatant was collected and incubated with 10 μmol·L^-1^ Bio-α-hederin or 10 μmol·L^-1^ D-biotin for 12 h. Subsequently, the mixture was incubated with streptavidin magnetic beads (Beyotime, Shanghai, China, P5085) at 4 °C for 2 h and then washed thrice with washing buffer. The proteins bound to the beads were harvested, separated by SDS-PAGE, and detected by western blotting.

### Patient sample collection

Seven samples of paired CRC tumor tissues and adjacent normal tissues were collected. Patient consent was obtained, and the study was approved by the Ethics Committee of the Affiliated Hospital of Nanjing University of Chinese Medicine (Approval Number: 2023NL-003-02) prior to commencement. The clinical characteristics of the patients are summarized in **Table 1**.

### Lentiviral transduction

To generate stable USP5 knockdown and overexpression cell lines, lentiviruses expressing USP5 small hairpin RNA (shRNA) and lentiviruses overexpressing USP5 were constructed by Tsingke Biotech (Beijing, China) and used to infect HCT116 cells. The target sequences of USP5 shRNA and the corresponding negative control shRNA (shNC) are shown in** Supplementary Table S2**, and the USP5-overexpressing (OE) target sequence is listed in **Supplementary Table S3**. HCT116 cells in the logarithmic growth phase were infected by adding 200 μL 1 × 10^8^ TU·mL^-1^ lentivirus in a six-well plates with 2 × 10^5^ cells per well combined with 5 μg·mL^-1^ polybrene. Stable transfected cells were positively selected with 2 μg·mL^-1^ puromycin. After 72 h, infection efficiency was assessed using qPCR and western blotting, respectively.

### Cell proliferation assays

For the CCK8 assay, cells were seeded at a density of 2 × 10^3^ cells/well in 96-well plates, 10% CCK8 (Beyotime, Shanghai, China, C0037) was added to each well after 1, 2, 3, 4, and 5 days of cell culture, and absorbance was measured at 450 nm after 1.5 h of incubation using a microplate reader (TECAN SPARK 10M, TECAN, Männedorf, Switzerland). For the colony formation assay, 1 × 10^3^ cells/well were plated in six-well plates and cultured for 2 weeks. Colonies were fixed with 4% PFA, stained with crystal violet, and counted.

### Statistical analysis

Data were analyzed using GraphPad Prism 9.5 (San Diego, CA, USA) and presented as mean ± standard deviation (SD). Student's *t*-test between two groups and one-way or two-way analysis of variance (ANOVA) followed by the Bonferroni post hoc test for multiple comparisons were used for statistical analyses. Statistical significance was set at *p* < 0.05.

## Results

### α-hederin showed anti-inflammatory effects *in vitro*

**Figure 1:**
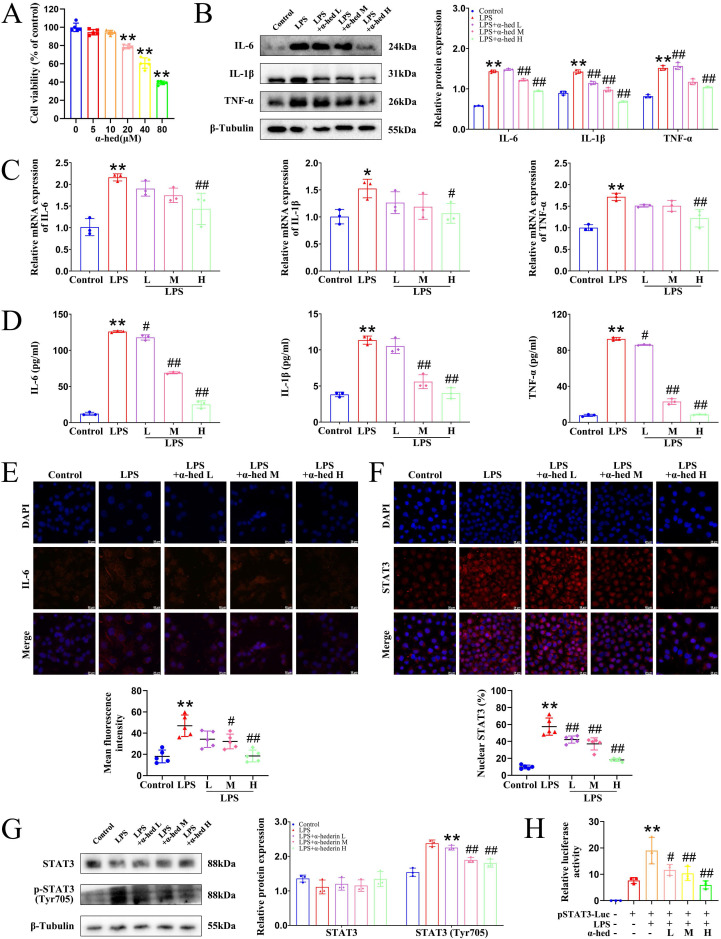
α-hederin showed anti-inflammatory effects *in vitro*. (A) Cell viability of normal human IEC NCM460 treated with α-hederin for 24 h at the indicated concentrations, n = 5. (B, C) The protein (B) and mRNA (C) expression levels of IL-6, IL-1β, and TNF-α were detected by western blotting and qPCR in LPS induced enteritis model of NCM460 cells after α-hederin treatment for 24 h, n = 3. (D) The concentration of IL-6, IL-1β, and TNF-α in the supernatant was detected using ELISA assays in LPS induced enteritis model of NCM460 cells after α-hederin treatment for 24 h, n = 3. (E) IF analysis of IL-6 in LPS induced enteritis model of NCM460 cells after α-hederin treatment for 24 h, n = 5. Scale bar = 20 μm. (F) Nuclear distribution of STAT3 in LPS induced enteritis model of NCM460 cells after α-hederin treatment for 24 h was analyzed by IF analysis, n = 5. Scale bar = 20 μm. (G) The expression of STAT3 and the levels of phosphorylated STAT3 were detected by western blotting, n = 3. (H) The transcriptional activity of STAT3 was detected by luciferase assay, n = 3. Data are presented as means ± SD. *p < 0.05, **p < 0.01 versus control group,^ #^*p* < 0.05, ^##^*p* < 0.01 versus LPS group.

To investigate the anti-inflammatory effects of α-hederin *in vitro*, first, we used the MTT assay to comprehensively determine the cytotoxicity of α-hederin on two normal human colonic epithelial cells, NCM460 and FHC, after treatment for 24 and 48 h. The two normal IECs exhibited tolerance to α-hederin treatment, with the half-maximal inhibitory concentration (IC_50_) value of 56.44 μmol·L^-1^ for NCM460 after 24 h of treatment (**Fig. 1A**), and 51.09 μmol·L^-1^ after 48 h of treatment (**Supplementary Fig. S1A**), respectively, and with IC_50_ value of 52.23 μmol·L^-1^ for FHC after 24 h of treatment (**Supplementary Fig. S1B**), and 50.91 μmol·L^-1^ after 48 h of treatment (**Supplementary Fig. S1C**), respectively. This indicated that α-hederin had low toxicity to normal human IECs. To reduce the toxic impact on NCM460 cells, the classical cell for studying inflammation in IECs, the subsequent low, medium, high concentrations of α-hederin were set as 2.5, 5, and 10 μmol·L^-1^, respectively, for 24 h. As LPS stimulation can greatly increase the level of inflammatory factors in NCM460 cells, 2 μg·mL^-1^ of LPS was used to induce cellular inflammation to mimic the inflammatory state 30. The experimental data from western blotting (**Fig. 1B**), qPCR (**Fig. 1C**), ELISA (**Fig. 1D**), and IF (**Fig. 1E**) assays showed that the expression of cellular inflammatory cytokines was significantly increased in the LPS group, revealing that the inflammatory model was established. When α-hederin was applied to the LPS-treated cells, the expression and concentration of IL-6, IL-1β, and TNF-α that had been substantially increased by LPS stimulation were significantly suppressed and restored to near normal levels. This meant that α-hederin could effectively inhibit the inflammatory response induced by LPS, revealing its intrinsic anti-inflammatory properties. IL-6 upregulation activates STAT3 and exacerbates the inflammatory response 31. In its inactivated state, STAT3 is predominantly found in the cytoplasm, and its activation is dependent on phosphorylation and translocation to the nucleus 16. Therefore, we examined the entry of STAT3 into the nucleus, and IF results showed that in LPS group, the distribution in the nucleus and the expression of STAT3 were notably increased compared to control group, whereas α-hederin intervention evidently decreased its distribution in nucleus and expression (**Fig. 1F**), which showed that α-hederin had anti-inflammatory effects *in vitro*. To further assess the activation of STAT3, the levels of phosphorylated STAT3 was also detected, and the immunoblotting result showed that α-hederin inhibited the increased STAT3 phosphorylation at Tyr705 in LPS group (**Fig. 1G**). As STAT3 is a transcription factor and above results revealed that α-hederin was a STAT3 inhibitor, the transcriptional activity of STAT3 was also detected using a STAT3-derived luciferase assay. As shown in **Fig. 1H**, α-hederin suppressed the increased STAT3 luciferase activity of NCM460 cells stimulated with LPS. In addition, nuclear factor-κappa B (NF-κB), another transcription factor, also plays an important role in inflammation. Therefore, we tested both at the same time to see if α-hederin specifically inhibited STAT3 using immunoblotting and luciferase assays, and determined that α-hederin failed to inhibit the increased NF-κB phosphorylation at Ser536 (**Supplementary Fig. S2A**) and the increased NF-κB luciferase activity in LPS group (**Supplementary Fig. S2B**). Therefore, α-hederin inhibited inflammation was associated with inhibition of STAT3 activity.

### α-hederin inhibited tumorigenesis, protected the intestinal barrier function, and reduced intestinal inflammation* in vivo*

**Figure 2:**
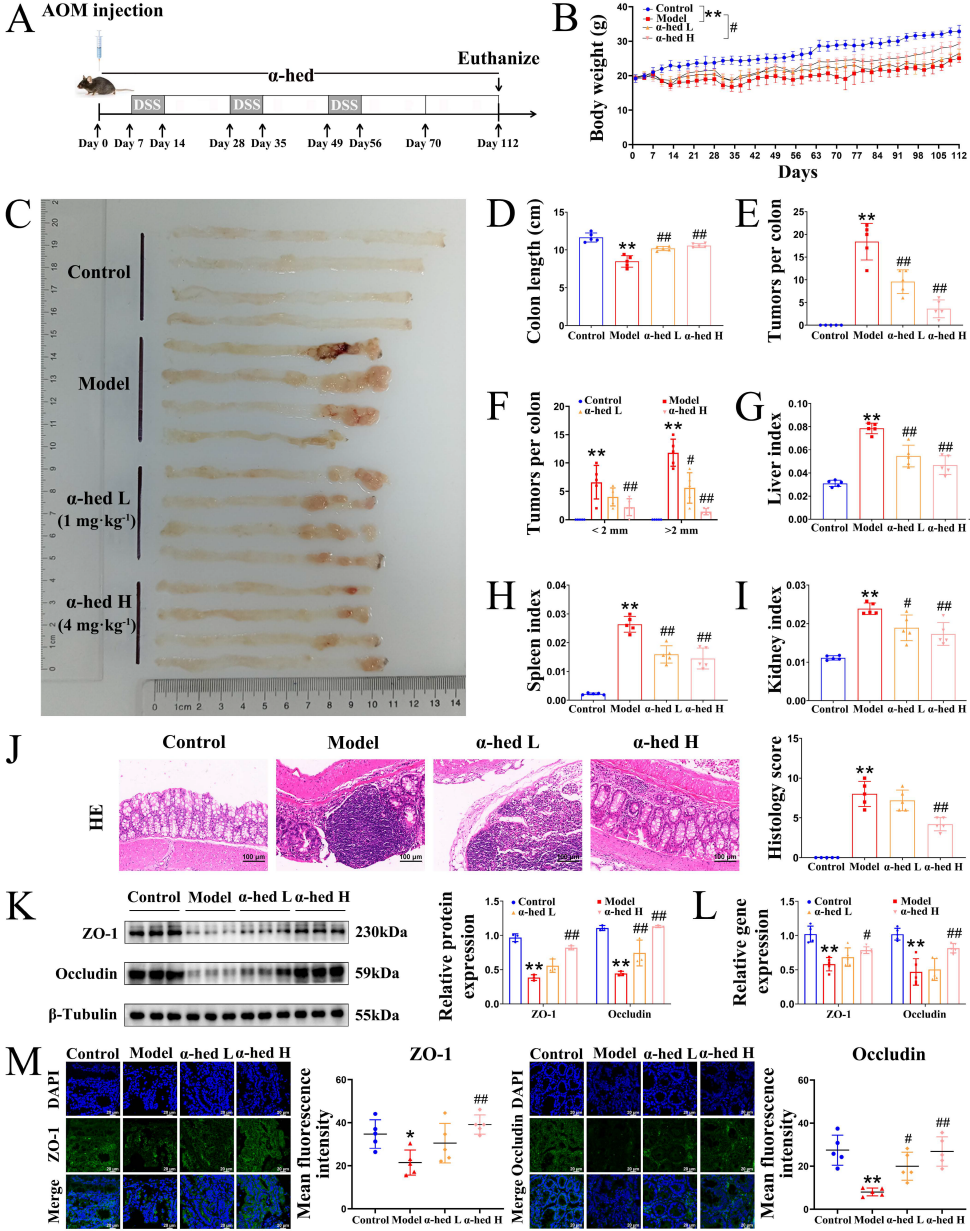
α-hederin inhibited tumor progression and improved the expression of TJ proteins associated with intestinal barrier function in CRC mice. (A) The modeling method of AOM/DSS-induced CRC in mice. (B) The body weight of mice, n = 5. (C) Tumors inside the colorectum of mice were photographed. (D) The length of the mouse colon, n = 5. (E) Statistical graph of a total number of tumors, n = 5. (F) Statistical graph of a total number of tumors diameter larger than 2 mm and smaller than 2 mm, n = 5. (G-I) The index of liver (G), spleen (H), and kidney (I) of mice, n = 5. (J) H&E staining of the colon and histology score, n = 5. Scale bar = 100 μm. (K, L) The protein (K) and mRNA (L) expression of ZO-1 and Occludin in colon tissues were detected by western blotting, n = 3, and qPCR, n = 5. (M) The expression of ZO-1 and Occludin in colon tissues were detected by IF analysis, n = 5. Scale bar = 20 μm. Data are presented as means ± SD. *p < 0.05, **p < 0.01 versus control group,^ #^*p* < 0.05, ^##^*p* < 0.01 versus model group.

Inflammation promotes the development and progression of colorectal tumors 2. As α-hederin had an inhibitory effect on inflammation (**Fig. 1**), and based on the fact that our previous studies showed that it inhibits the malignant transformation of IECs 12, 13, we further investigated the role and mechanism of α-hederin on the transformation of colitis carcinoma in AOM/DSS-induced CRC-mice, the model of which is a process from chronic intestinal inflammation to CRC, and mimics the course of inflammatory bowel disease patients to CRC patients 32. A schematic representation of the mice model was shown in **Fig. 2A**. The body weight of the mice in the model group decreased rapidly during DSS treatment and gradually recovered when treated with water. At week 16, the body weight in model group was lower than that in control group, whereas the body weight of mice in α-hederin H group exhibited elevated body weight compared with that in model group (**Fig. 2B**). Compared to the control group, the colon length of mice in the model group was significantly shortened (**Fig. 2D, Supplementary Fig. S3**), and widespread tumors were identified in the colon tissue (**Fig. 2C**), with a significant increase in the total tumor number (**Fig. 2E**) and the number of tumors greater than 2 mm in diameter (**Fig. 2F**) in the model group. Whereas, α-hederin treatment effectively reversed this phenomenon, indicating that AOM/DSS-induced CRC-mice model has been successfully established, but α-hederin inhibited tumor growth in CRC-mice. Moreover, the ratios of the liver, spleen, kidney, and body weight were higher in the model group than in the control group. After treatment with α-hederin, these indexs of liver, spleen, and kidney were both lower than those recorded in model mice (**Fig. 2G, H, I**).

In addition, H&E staining showed that the intestinal epithelium of the colorectal tissue of control mice was intact, with a regular arrangement of glands, normal crypts, and no inflammatory cell infiltration. In contrast, the epithelial cells in the model group lost polarity, were nested and sieve-shaped, and necroses were observed in the center. The epithelial mucus layer disappeared, the nucleus-to-cytoplasm ratio increased, nuclear staining was deep, the nucleolus and nuclear schizophrenia phenomenon and inflammatory cell infiltration were obvious, and the histopathological impairment was severe with a high histology score. However, the above phenomena were significantly relieved and the histology score was reduced in α-hederin group (**Fig. 2J**), which confirmed the effectiveness of α-hederin in alleviating colorectal adenoma carcinogenesis.

The expression of tight junction (TJ) proteins such as zonula occludens-1 (ZO-1) and Occludin is downregulated during tumorigenesis 33. The protein and mRNA expression in colon tissues were examined using western blotting (**Fig. 2K**) and qPCR (**Fig. 2L**), and the results showed that α-hederin restored the downregulated expression of TJ proteins in AOM/DSS treated mice. In line with this, IF results also showed that both ZO-1 and Occludin protein expression were downregulated in model group compared with control group, which was reversed by α-hederin (**Fig. 2M**), indicating that α-hederin protected the integrity of the intestinal barrier in CRC-mice.

**Figure 3:**
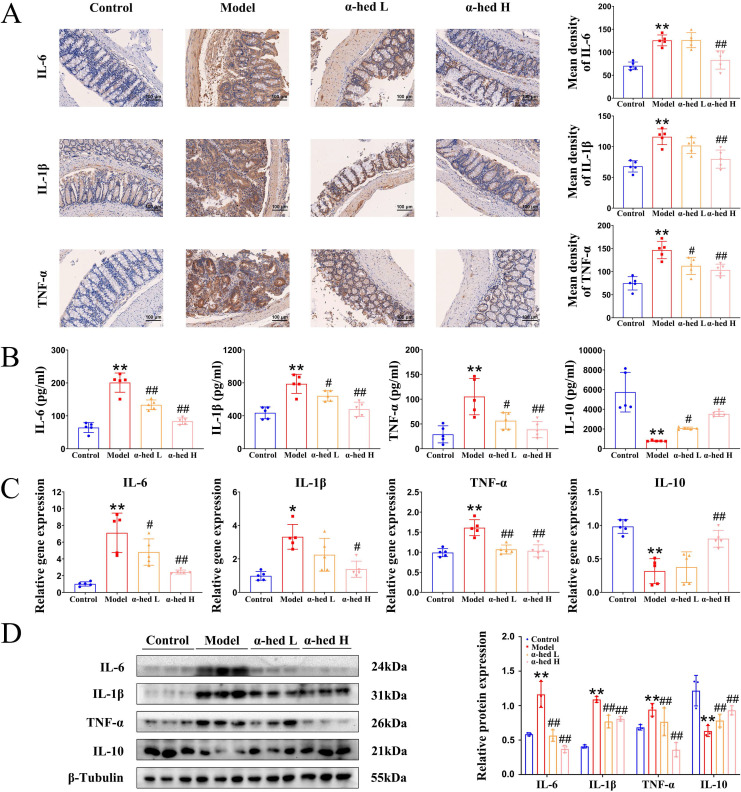
α-hederin inhibited the expression of IL-6, IL-1β, TNF-α and increased the expression of IL-10 in CRC mice. (A) IHC staining of IL-6, IL-1β, TNF-α protein and statistics in colon, n = 5. Scale bar = 100 μm. (B) The content of cytokines IL-6, IL-1β, TNF-α, and IL-10 in the serum of mice, n = 5. (C, D) The mRNA (C) and protein (D) expression levels of cytokines IL-6, IL-1β, TNF-α, and IL-10 in colon tissues of mice were detected by qPCR, n = 5, and western blotting, n = 3. Data are presented as means ± SD. *p < 0.05, **p < 0.01 versus control group,^ #^*p* < 0.05, ^##^*p* < 0.01 versus model group.

CRC progresses from inflammation to cancer in a stepwise manner. Inflammation, an important promoter of both tumor initiation and progression 2, is a major risk factor for CRC development, and patients with inflammatory bowel disease have a high risk of CRC 34. Disrupted epithelial TJ proteins are major contributors to intestinal barrier dysfunction and gut inflammation. Excessive exposure to pro-inflammatory mediators disrupts TJ proteins and increases intestinal permeability 35. So, we detected the expression of inflammatory cytokines IL-6, IL-1β, and TNF-α by IHC analysis. As shown in** Fig. 3A**, IL-6, IL-1β, and TNF-α expression had a significant enhancement in the intestinal tract from mice in model group as compared to control group, whereas α-hederin induced significant inhibition of these inflammatory cytokines expression. To confirm these findings, ELISA assays were conducted to detect the expression of IL-6, IL-1β, TNF-α, and anti-inflammatory cytokine IL-10 in mice serum, and the result showed that the increased inflammatory cytokines IL-6, IL-1β, TNF-α in the serum of AOM/DSS treated mice were decreased after α-hederin treatment, but the decreased anti-inflammatory cytokine IL-10 in the serum of AOM/DSS treated mice was increased after α-hederin treatment (**Fig. 3B**). In line with this, qPCR (**Fig. 3C**) and western blotting (**Fig. 3D**) in colon tissues of mice further evaluated the anti-inflammatory effects of α-hederin. Taken together, α-hederin effectively inhibited tumor-associated inflammation and the development of CRC.

### α-hederin inhibited the activation of the p38 mitogen-activated protein kinase (MAPK)/STAT3 inflammatory-cancer transformation pathway and attenuated oxidative stress

**Figure 4:**
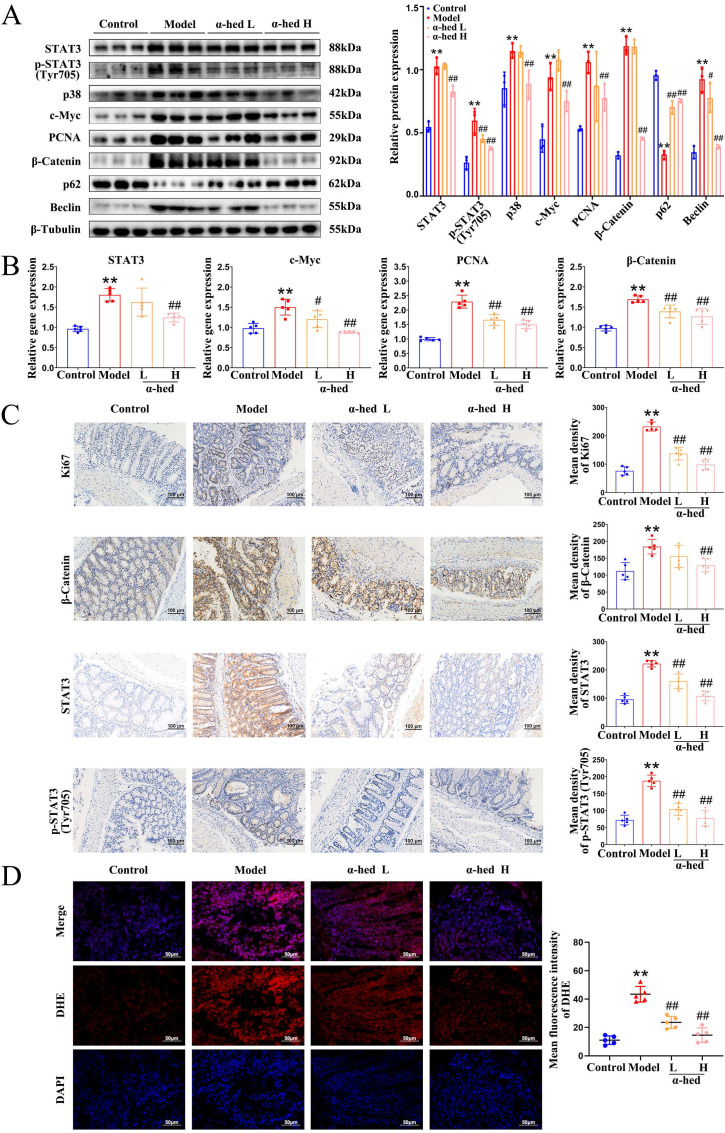
The effect of α-hederin on the expression of autophagy-related proteins and proliferation-related proteins. (A) The protein expression level of STAT3, p-STAT3 (Tyr705), p38, c-Myc, PCNA, β-Catenin, p62, and Beclin in colon tissues of mice were detected by western blotting, n = 3. (B) The mRNA expression level of STAT3, c-Myc, PCNA, and β-Catenin in colon tissues of mice were detected by qPCR, n = 5. (C) IHC staining of Ki67, β-Catenin, STAT3 and phosphorylated STAT3 protein and statistics in colon tissues of mice, n = 5. Scale bar = 100 μm. (D) Representative IF images of ROS in colon tissues of mice, n = 5. Scale bar = 50 μm. Data are presented as means ± SD. **p < 0.01 versus control group,^ #^*p* < 0.05, ^##^*p* < 0.01 versus model group.

IL-6 is an important tumor-initiating factor in CRC, and the proliferative and survival effects of IL-6 are primarily mediated by the downstream transcription factor STAT3 36, which is the forward feedback loop of STAT3 that promotes cancer malignancy. The upregulation of IL-6 leads to STAT3 hyperactivation 37, constituting a positive feedback loop that amplifies and maintains STAT3 signaling. Our study found that α-hederin inhibited the high expression of IL-6 in colon tissues of AOM/DSS treated mice (**Fig. 3**), we then detected the expression of STAT3 in colon tissues, and determined that both STAT3 expression and phosphorylation were increased in CRC tissues (**Fig. 4A**), which may be the result of a forward feedback loop induced by STAT3 that amplified STAT3 signaling and conferred a degree of malignancy to the inflammatory cancer transformation of IECs. But α-hederin had the negative effect on total STAT3 protein expression, and decreased STAT3 phosphorylation at Tyr705 (**Fig. 4A**), indicating that α-hederin inhibited STAT3 activation and reduced STAT3 activity* in vivo*, which was consistent with* in vitro* results (**Fig. 1**). Considering that STAT3 was a transcription factor, we also detected its mRNA level and found that STAT3 mRNA expression was increased in AOM/DSS treated mice, but decreased in α-hederin group (**Fig. 4B**), which was consistent with protein level results (**Fig. 4A**), indicating that α-hederin inhibited STAT3 transcriptional and translational activities.

The p38 MAPK pathway is essential for proliferation and stress response, is activated by extracellular stimuli such as oxidative stress, growth factors, and inflammation, and plays an important role in CRC 38, 39. Specifically, inhibition of the p38 MAPK pathway alleviates CRC. The p38 MAPK and STAT3 pathways are prominent stress-responsive pathways that regulate cell survival and proliferation during CRC development. We further found that p38 levels were increased in model group, and decreased in α-hederin H group (**Fig. 4A**). Infinite cell proliferation is a hallmark of cancer. c-Myc is a proto-oncogene, transcription factor, and cell cycle-promoting factor regulated by p38 MAPK 40 and is a downstream target of STAT3 41. The colon tissues from the model group mice showed higher levels of c-Myc, and the proliferation marker PCNA, both of which were decreased in α-hederin group (**Fig. 4A, B**).

Correspondingly, Ki67 staining showed that cell proliferation in colorectal tissue was significantly inhibited with α-hederin (**Fig. 4C**). As β-catenin is an activator of the STAT3 signaling 42, and is activated by oxidative stress 43, we next performed western blotting, qPCR and IHC assays, and the results showed that β-catenin was upregulated in AOM/DSS treated mice and downregulated in α-hederin group (**Fig. 4A, B, C**). IHC analysis also revealed that administration of α-hederin reversed AOM/DSS-induced STAT3 and phosphorylated STAT3 expression (**Fig. 4C**), indicating that α-hederin suppresses the development of colitis-associated tumors.

Activated p38 MAPK/STAT3 signaling is required for the induction of autophagy 44-46, which enhances the proliferation and survival of tumor cells by supplying nutrients. Autophagy is induced by TNF-α, and IL-6, whereas blocked by IL-10, and also triggers the secretion of TNF-α, and IL-6 47. Sequestosome 1 (p62) and Beclin are important proteins involved in intracellular autophagy. p62 is a classical autophagy receptor, and the level of intracellular p62 reflects autophagic activity. When autophagic activity is enhanced, the intracellular level of p62 decreases; conversely, when autophagic activity is inhibited, the intracellular level of p62 increases 48. It was reported that the increased expression of p62 inhibited inflammasome activation and IL-1β production 49, and prevented cellular overinflammation under persistent oxidative stress 50, and suppressing the overactivation of Beclin mediated autophagy could reduce the inflammation level 51. Therefore, we further examined the expression levels of autophagy proteins in colon tissues of mice and determined that the protein expression level of Beclin was significantly higher in the colon tissue of the model group than in the control group, whereas p62 decreased (**Fig. 4A**), consistent with previous reports that the protein expression level of the autophagy marker Beclin was increased in tumor tissues than in paired adjacent normal tissues of patients with CRC, and autophagy inhibitor p62 was decreased in colon tissues of AOM/DSS-induced CRC mice 52. However, α-hederin reversed the decreased expression of p62 and the increased expression of Beclin in CRC-mice. Therefore, these results revealed that p38 MAPK/STAT3 signaling was activated and autophagy was subsequently induced in the progression of CRC, leading to a positive feedback loop that amplified inflammation, however, these phenomena were reversed by α-hederin.

Furthermore, ROS are highly reactive oxygenated substances that can activate the p38 MAPK pathway 44, which in turn regulates cellular inflammatory responses. However, excessive ROS levels lead to oxidative stress, exacerbate the inflammatory response, induce DNA mutations 53, contribute to tumor promotion and progression 54, and induce autophagy 55. Our results revealed that compared with control group, colon tissues of model group exhibited more ROS, but α-hederin reduced ROS production in colon tissues of CRC-mice (**Fig. 4D**). These results suggested that α-hederin inhibited inflammatory-cancer transformation by inhibiting p38 MAPK/STAT3 signaling and attenuated oxidative stress.

### α-hederin inhibited the binding of p62 with STAT3 followed by inhibition of STAT3 phosphorylation at Tyr705

**Figure 5:**
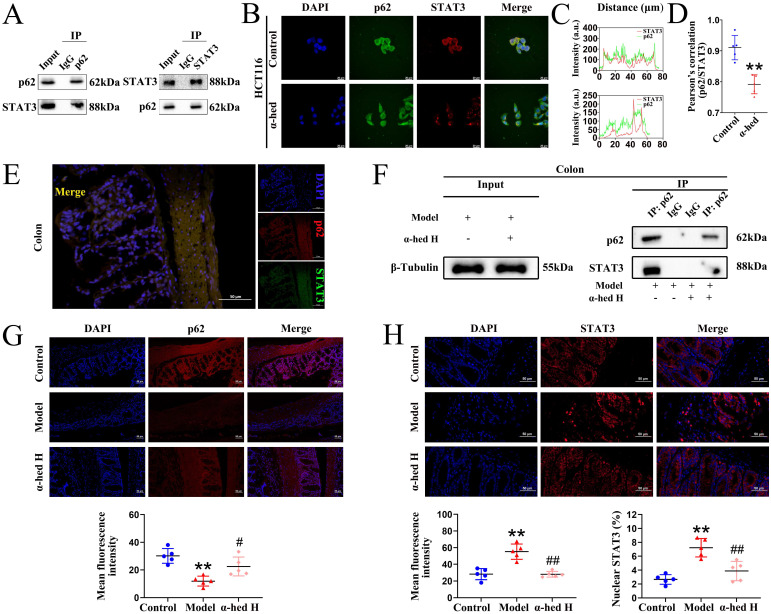
Inhibition of p62 binding with STAT3 by α-hederin inhibited STAT3 entry into the nucleus to undergo phosphorylation. (A) The Co-IP experiments were performed to validate the protein interactions between STAT3 and p62 in HCT116 cells by using p62 antibody and STAT3 antibody separately. (B) Co-localization of STAT3 with p62 in HCT116 cells treated with or without α-hederin using confocal observation, and the qualitative (C) and quantitative (D) analysis of co-localization were performed with Image J, n = 5. Scale bar = 20 μm. (E) Co-localization of STAT3 with p62 in colon tissue of mice in control group. Scale bar = 50 μm. (F) The Co-IP experiment was performed to validate α-hederin inhibition of p62 binding to STAT3 in colon tissues of mice in model and α-hederin H groups. (G) IF analysis of p62 in colon tissues of mice, n = 5. Scale bar = 50 μm. (H) IF images showing STAT3 expression level and nuclear translocation in colon tissues of mice, n = 5. Scale bar = 50 μm. Data are presented as means ± SD. **p < 0.01 versus control group,^ #^*p* < 0.05, ^##^*p* < 0.01 versus model group.

As a transcription factor, STAT3 is transported from the cytoplasm to the nucleus to activate transcription, and its activity is regulated by phosphorylation 16. Recent studies have shown that p62 regulates STAT3 phosphorylation 56. Our previous results revealed that p62 protein expression negatively correlated with STAT3 protein expression (**Fig. 4A**). Therefore, we speculated that some modulation may exist between the two proteins and aimed to understand the impact of α-hederin on the modulation between p62 and STAT3. First, we performed Co-IP experiments on HCT116 cells and colon tissues. The results in HCT116 cells showed that the proteins recruited by the p62-beads contained STAT3, and the proteins recruited by the STAT3-beads contained p62, validating the physical interaction between p62 and STAT3 (**Fig. 5A**). Then, we tested the cytotoxic effects of α-hederin on HCT116 cells using MTT assay, and found that α-hederin significantly inhibited the growth of HCT116 cells in a concentration- and time-dependent manner, and the IC_50_ of α-hederin in HCT116 cells was 15.69 μmol·L^-1^ for 24 h (**Supplementary Fig. S4A**), and 12.45 μmol·L^-1^ for 48 h (**Supplementary Fig. S4B**). Notably, NCM460 and FHC exhibited higher tolerance to α-hederin treatment (**Fig. 1A, Supplementary Fig. S1**), which indicated that α-hederin has low toxicity to human normal IECs. The subsequent studies of HCT116 cells treated with low, medium, and high concentrations of α-hederin set as 5, 10, and 20 μmol·L^-1^, respectively, for 24 h. Confocal microscopy results also revealed that p62 co-localized with STAT3 (**Fig. 5B**), and the co-localization qualitative (**Fig. 5C**) and quantitative (**Fig. 5D**) analysis using ImageJ confirmed the strong spatial co-expression of p62 with STAT3. However, α-hederin weakened the interaction between p62 and STAT3 (**Fig. 5B, C, D**). Correspondingly, the interaction between p62 and STAT3 was also observed in the colon tissues of mice in control group (**Fig. 5E**), and compared with model group, the mutual binding of p62 and STAT3 in colon tissues of mice in α-hederin H group was inhibited (**Fig. 5F**) and STAT3 phosphorylation at Tyr705 was attenuated simultaneously (**Fig. 4A, C**), indicating that α-hederin increased the expression of anti-inflammatory p62 protein 49, 50 in colon tissues of model group (**Fig. 4A, 5G**), as well as inhibited mutual binding of p62 and STAT3, thereby reducing STAT3 translocation from the cytoplasm to the nucleus (**Fig. 4C, 5H**). This may have resulted in the inhibition of STAT3 phosphorylation at Tyr705 followed by the inhibition of its constitutive activation (**Fig. 4A, C**) in colon tissues of AOM/DSS treated mice.

### USP5 was a direct target of α-hederin

**Figure 6:**
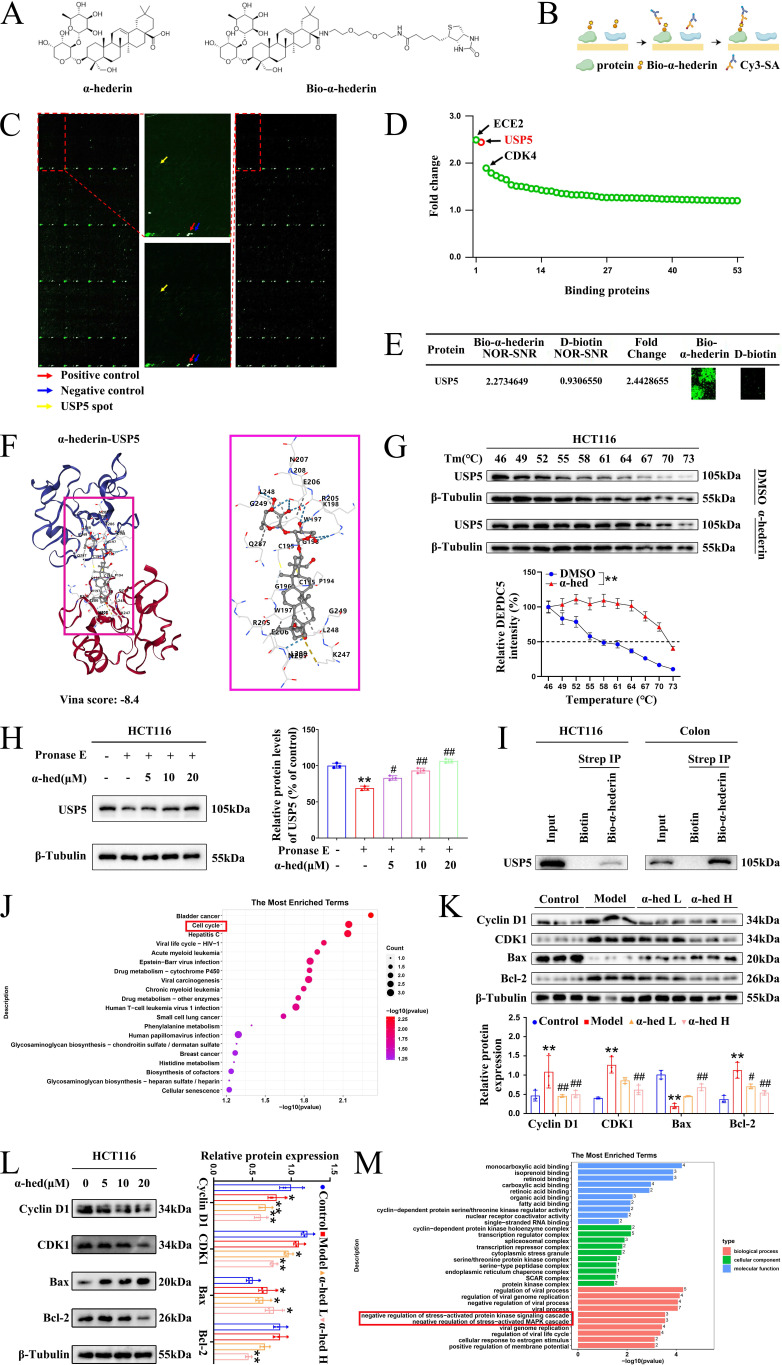
Identification of α-hederin binding proteins. (A) Chemical structure of α-hederin and Bio-α-hederin. (B) Schematic showing steps for identifying α-hederin binding proteins using microarrays fabricated with recombinant human proteins. (C) Representative image of protein array showing positive (red arrow), negative control (blue arrow) spots, and spots for USP5 (yellow arrow). The left panel shows the microarray scan of Bio-α-hederin, the right panel shows the microarray scan of D-biotin, and the center panel shows the partial enlargement of the microarray of Bio-α-hederin (top) and D-biotin (bottom), respectively. (D) The top 3 potential binding targets of α-hederin were identified by proteome microarray. (E) Magnified image of Bio-α-hederin and D-biotin binding to USP5 spot on the protein array. The values of NOR-SNR and FC were showed. (F) Three- and two-dimensional docking of α-hederin and USP5. (G) CETSA was performed to assess the effect of α-hederin on the thermal stability of USP5 in HCT116 cells, n = 3. (H) DARTS assay measured the impact of α-hederin on the proteolytic stability of USP5 in HCT116 cells, n = 3. (I) Streptavidin‒biotin pull-down assay was performed on the protein mixture extracted from HCT116 cells and colon tissues of CRC-mice. (J) Pathway analysis of potential proteins in the KEGG database. (K, L) The effects of α-hederin treatment on cell proliferation, apoptosis, and related processes through *in vivo* (K) and *in vitro* (L) experiments. (M) GO enrichment analysis of the proteins interacting with α-hederin based on the GO database. Data are presented as means ± SD. *p < 0.05, **p < 0.01 versus DMSO group or control group, ^#^*p* < 0.05, ^##^*p* < 0.01 versus pronase E group or model group.

To precisely explore the molecular mechanism through which α-hederin exerted its anti-CRC activity, we synthesized a biotin-labeled α-hederin probe (Bio-α-hederin) (**Fig. 6A**). The synthesis and purification of Bio-α-hederin were described in **Supplementary Fig. S5**. Next, we performed a proteome microarray assay using HuProt^TM^ V4.0 21K human proteome microarray by incubating with Bio-α-hederin or D-biotin (**Fig. 6B, C**). Notably, USP5 produced a robust NOR-SNR of 2.2734649 and a high FC of 2.4428655 (**Fig. 6C, D, E**), indicating that USP5 may be a direct pharmacological target of α-hederin. USP5 piqued our interest because it plays a crucial role in promoting malignant tumor progression and exacerbating the inflammatory response 25. However, there are few reports on whether USP5 is involved in the development of CRC, and the function and regulatory network of USP5 in CRC need to be further explored in depth, whereas the association between α-hederin and USP5 has not yet been reported in relevant studies. To validate the interaction of α-hederin with USP5 protein, we employed molecular docking (**Fig. 6F**), the USP5 protein was assigned as the receptor and α-hederin was assigned as the ligand. The vina scores of α-hederin with USP5 was -8.4, indicating that α-hederin had strong binding activity with USP5. Furthermore, we verified the intracellular binding of USP5 to α-hederin using CETSA experiment in HCT116 cells and determined that the thermal stability of USP5 increased in the presence of α-hederin (10 μmol·L^-1^) compared with the control group (DMSO) (**Fig. 6G**). Correspondingly, the DARTS assay in HCT116 cells showed that α-hederin prevented Pronase E-induced USP5 proteolysis (**Fig. 6H**). Subsequently, we used streptavidin-biotin pull-down assays and determined that Bio-α-hederin captured USP5 in both HCT116 cells and colon tissues of CRC-mice (**Fig. 6I**). In summary, these data confirmed that USP5 was a direct binding protein of α-hederin.

Based on the pathway analysis of these potential positive binding proteins of α-hederin in the Kyoto Encyclopedia of Genes and Genomes (KEGG) database, we found that they were significantly enriched in the cell cycle pathway, which is closely associated with cell proliferation (**Fig. 6J**), subsequently, we further validated the effects of α-hederin on cell proliferation, apoptosis, and related processes through *in vivo* and *in vitro* experiments. The *in vivo* experiment result showed that α-hederin decreased the high expression levels of proliferation-related molecules Cyclin D1 and CDK1 in colon tissues of AOM/DSS treated mice (**Fig. 6K**), which was consistent with the effects of α-hederin on the expression of PCNA, c-Myc and on the number of Ki67-positive cells (**Fig. 4A, B, C**). Notably, α-hederin treatment also increased the expression of pro-apoptotic protein Bax and decreased the expression of anti-apoptotic protein Bcl-2 in AOM/DSS group (**Fig. 6K**). Accordingly, the *in vitro* experimental results showed that the expression levels of Cyclin D1, CDK1, and Bcl-2 decreased and Bax increased in HCT116 cells after α-hederin treatment (**Fig. 6L**). These results suggested that α-hederin treatment inhibited colorectal tumor cells proliferation and promoted their apoptosis *in vivo* and *in vitro*. In addition, the proteins interacting with α-hederin primarily involved in negative regulation of stress-activated protein kinase signaling cascade and negative regulation of stress-activated MAPK cascade biological processes (**Fig. 6M**) based on Gene Ontology (GO) enrichment analysis, consistent with our experimental results in **Fig. 4**.

### USP5 was upregulated in CRC tissues and CRC cells and USP5-mediated CRC proliferation occurred through STAT3

**Figure 7:**
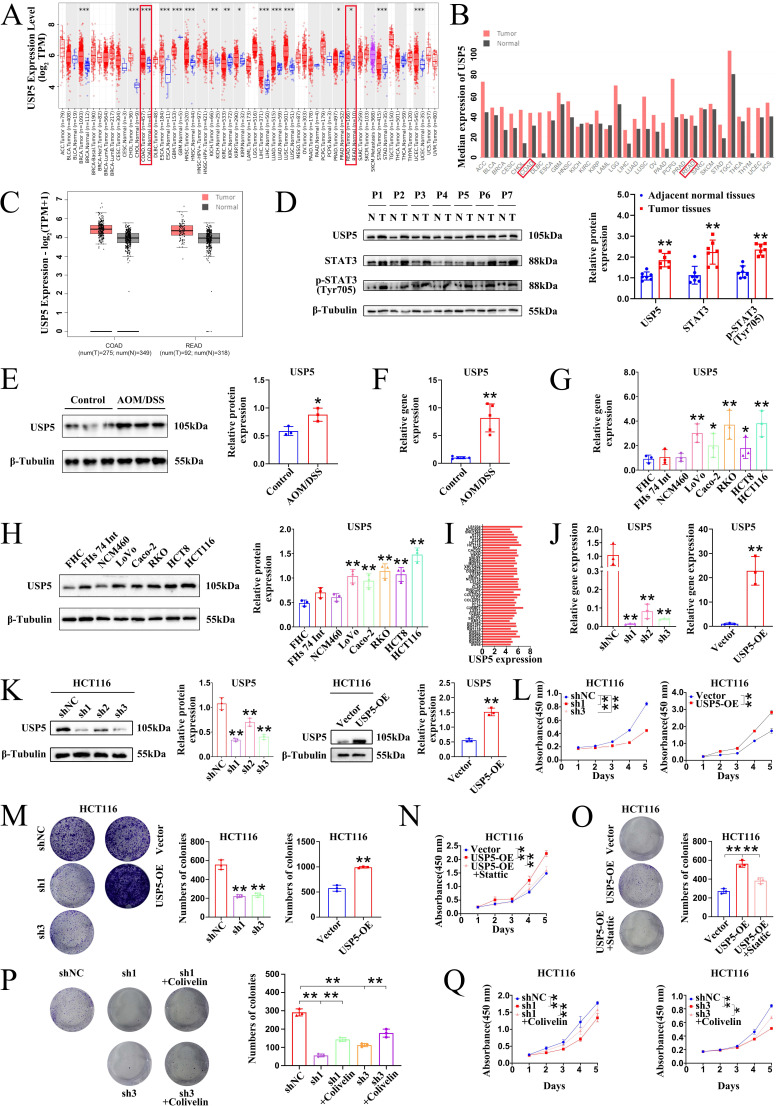
USP5 acted as a pro-cancer factor in CRC. (A) Differential gene expression of USP5 between tumor and adjacent normal tissues from TIMER database. (B) The bar plot of USP5 gene expression profile across all tumor samples and paired normal tissues from GEPIA database. (C) The gene expression of USP5 was analysed using the GEPIA database in datasets: COAD and READ. (D) The expression status of USP5, STAT3 and p-STAT3 (Tyr705) was simultaneously detected in paired CRC tumor tissues (T) and adjacent normal tissues (N) by immunoblotting, n = 7. (E, F) The protein and gene expression of USP5 in colon tissues of mice in control group and AOM/DSS group were detected by western blotting (E), n = 3, and qPCR (F), n = 5. (G, H) The expression of USP5 was examined in normal human IECs and CRC cell lines by qPCR (G) and western blotting (H), n = 3. (I) The expression of USP5 in different CRC cell lines in the CCLE database. (J, K) The qPCR (J) and western blotting (K) analysis confirmed the knockdown or overexpression efficacy of USP5 in HCT116 cell lines, n = 3. (L) Cell viability was determined by CCK8 assay after USP5 knockdown or overexpression, n = 3. (M) The colony formation ability was evaluated after USP5 knockdown or overexpression, n = 3. (N, O) The growth ability (N) and colony formation ability (O) of USP5 overexpression cells was evaluated after treatment with STAT3 inhibitor Stattic, n = 3. (P, Q) The colony formation ability (P) and growth ability (Q) of USP5 knockdown cells was evaluated after treatment with STAT3 activator Colivelin, n = 3. Data are presented as means ± SD. *p < 0.05, **p < 0.01, ***p < 0.001.

USP5 is an important member of the USP family that promotes tumorigenesis by deubiquitinating and stabilizing oncoproteins 27. Binding of α-hederin to USP5 prompted us to address whether α-hederin inhibited inflammatory-cancer transformation by targeting USP5. To conduct a comprehensive analysis of USP5 expression in patients with colon adenocarcinoma (COAD) and rectum adenocarcinoma (READ), we first analyzed data from the Tumor Immune Estimation Resource (TIMER) database and determined that USP5 was highly expressed in COAD tumors (n = 457) compared with adjacent normal tissues (n = 41) and highly expressed in READ tumors (n = 166) compared with adjacent normal tissues (n = 10) (**Fig. 7A**). We also accessed the bar plot of USP5 gene expression profiles across all tumor samples and paired normal tissues leveraging the Gene Expression Profiling Interactive Analysis (GEPIA) database (**Fig. 7B, C**), uncovering its marked upregulation in 275 COAD tissues compared to 349 normal colon tissues, and its high expression in 92 READ tumor samples compared to 318 paired normal tissues, highlighting its potential role in colon carcinogenesis. To confirm our hypothesis, USP5 protein levels in seven paired CRC tissues were examined and were shown to be higher in CRC tissues than in adjacent normal tissues (**Fig. 7D**). Given that α-hederin retarded colorectal tumorigenesis progression was associated with inhibition of STAT3 activation (**Fig. 1, 4, 5**), STAT3 and phosphorylated STAT3 of paired CRC tissues were tested simultaneously, and results showed that they were also higher expressed in CRC tissues than paired normal tissues (**Fig. 7D**), which highlighted the indispensable role of USP5 and STAT3 in CRC development and progression.

For further validation, USP5 expression was measured *in vivo* and *in vitro*. As shown in **Fig. 7E, F,** the protein and gene expression of USP5 in the colon tissues of mice in the control and AOM/DSS groups were detected by western blotting and qPCR, and it was shown that USP5 was strongly expressed in the colon tissues of AOM/DSS-treated mice. In parallel, we measured USP5 expression in five human CRC cell lines (Lovo, Caco-2, RKO, HCT8, and HCT116) and three normal human IECs (FHC, FHs 74 Int, and NCM460) (**Fig. 7G, H**). Consistently, USP5 mRNA and protein expression levels were significantly higher in CRC cell lines than in normal IECs. In addition, the gene expression levels of USP5 in CRC cells in the Cancer Cell Line Encyclopedia (CCLE) database were determined to verify the qPCR and western blotting results (**Fig. 7I**). Collectively, these data provided compelling evidence for the significant upregulation of USP5 in CRC, indicating that USP5 acts as an oncogene in CRC pathogenesis and progression. To test this hypothesis, lentivirus-based HCT116 cells were used to stably silence or overexpress USP5, and the transfection efficiency was confirmed by qPCR (**Fig. 7J**) and western blotting (**Fig. 7K**). Both qPCR and western blotting results showed that sh1 exhibited the highest USP5 knockdown efficiency, and follow-up studies on USP5 knockdown were conducted using sh1. The results of the CCK8 assays showed that USP5 knockdown significantly impaired proliferation, whereas USP5 overexpression dramatically enhanced proliferation (**Fig. 7L**). Colony formation analysis further revealed that knockdown of USP5 markedly reduced colony formation in terms of both number and size compared with shNC cells, whereas overexpression of USP5 increased clone formation in terms of both number and size compared with vector cells (**Fig. 7M**). Therefore, USP5 may be a potential therapeutic target for CRC progression.

To further explore whether USP5-mediated CRC proliferation occurs through STAT3, rescue experiments were performed using USP5 stable cells. Inhibition of STAT3 using Stattic evidently suppressed the growth and colony formation ability of HCT116 cells overexpressing USP5 (**Fig. 7N, O**). Instead, activation of STAT3 using Colivelin notably elevated the colony formation and growth capacity of HCT116 cells knocking down USP5 (**Fig. 7P, Q**). In general, our results corroborated that USP5 promoted CRC proliferation through STAT3 activation.

### α-hederin decreased USP5 expression

**Figure 8:**
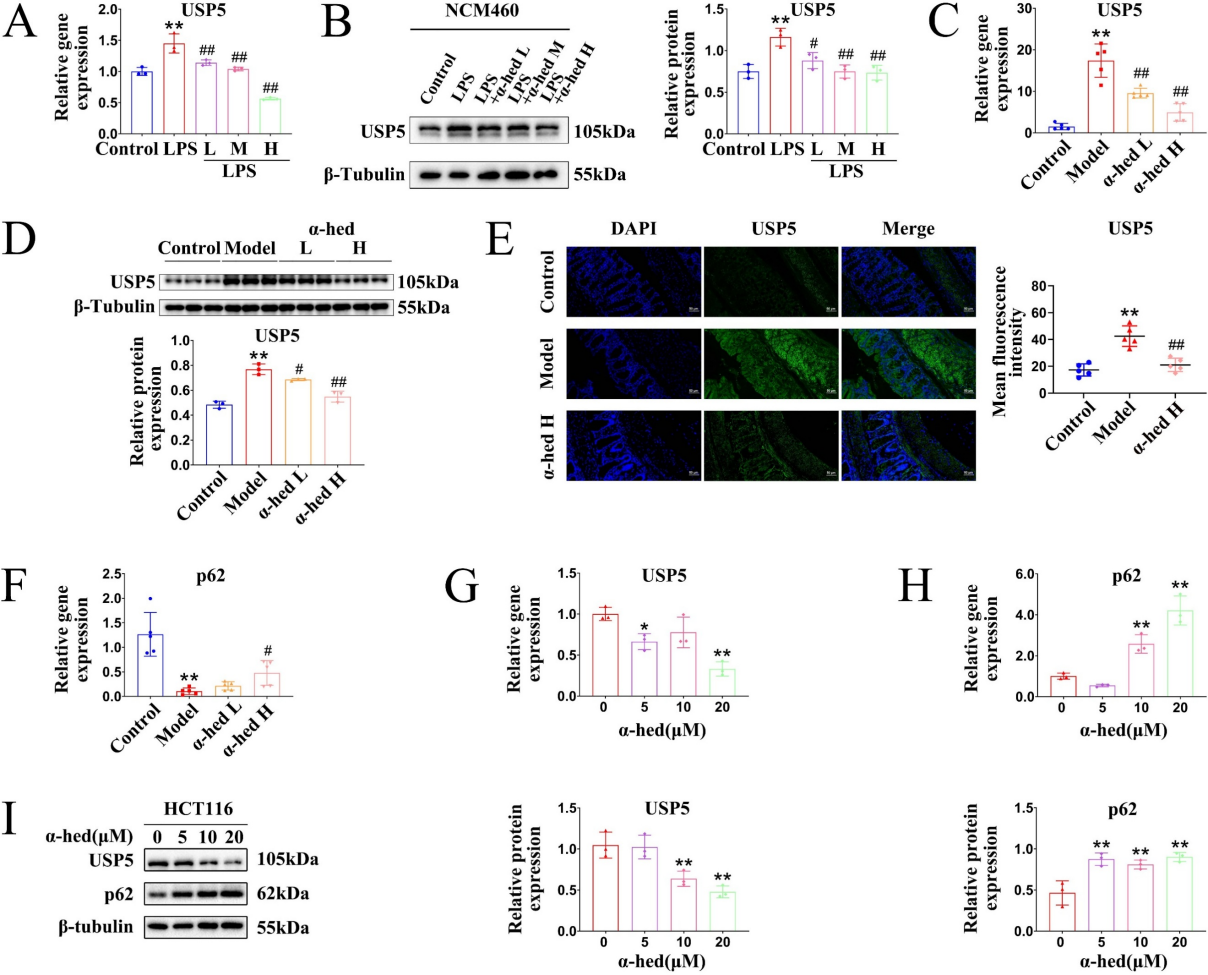
Effect of α-hederin on USP5 expression. (A, B) The mRNA and protein expression levels of USP5 were detected by qPCR (A) and western blotting (B) in LPS induced enteritis model of NCM460 cells after α-hederin treatment for 24 h, n = 3. (C, D) The mRNA and protein expression levels of USP5 in colon tissues of mice were detected by qPCR (C), n = 5, and western blotting (D), n = 3. (E) IF analysis of USP5 in colon tissues of mice, n = 5. Scale bar = 50 μm. (F) The mRNA expression level of p62 was detected by qPCR in colon tissues of mice, n = 5. (G) The mRNA expression level of USP5 was detected by qPCR in HCT116 cells treated with α-hederin for 24 h, n = 3. (H) The mRNA expression level of p62 was detected by qPCR in HCT116 cells treated with α-hederin for 24 h, n = 3. (I) USP5 and p62 protein expression levels were detected by western blotting in HCT116 cells treated with α-hederin for 24 h, n = 3. Data are presented as means ± SD. *p < 0.05, **p < 0.01 versus control group,^ #^*p* < 0.05, ^##^*p* < 0.01 versus LPS group or model group.

Given that USP5 may be a pro-oncogene in CRC (**Fig. 7**), which is a direct target of α-hederin (**Fig. 6**), we wondered whether the expression of USP5 could be regulated by α-hederin. Therefore, we conducted a series of experiments *in vitro* and* in vivo*. In the cellular inflammation model, USP5 was upregulated in LPS group, but was downregulated with α-hederin treatment both at mRNA and protein level (**Fig. 8A, B**). In AOM/DSS-induced CRC model, α-hederin reversed the upregulated mRNA and protein expression levels of USP5 in colon tissues of CRC-mice and was dose-dependent (**Fig. 8C, D**), which was also confirmed by IF experiments (**Fig. 8E**). As a ubiquitin-binding protein, p62 acts as a scaffolding protein through self-interactions and ubiquitin-binding domains, facilitating the formation and degradation of ubiquitinated aggregates 57. In contrast, DUBs, such as USP-associated proteins, reverse the effects of ubiquitination by removing the ubiquitin chain from the target proteins 21. As shown in **Fig. 4A, 5G**, p62 protein expression was decreased in the colon tissues of CRC mice, which could be reversed by α-hederin. However, USP5 mRNA and protein levels were increased in the colon tissues of CRC mice (**Fig. 7E, F**), which could be reversed by α-hederin (**8C, D, E**). We further detected the mRNA expression of p62 in colon tissues and found that its level was also decreased in model group, but was reversed by α-hederin (**Fig. 8F**). In CRC cell HCT116, we also observed that after α-hederin treatment, the mRNA and protein expression of USP5 were decreased, whereas those of p62 were increased (**Fig. 8G, H, I**), which corroborated with their role in the regulation of ubiquitination processes. These results revealed a negative correlation between p62 and USP5 expression, Interestingly, STAT3 is an ubiquitinated protein and is degraded dependent on the ubiquitin-proteasome system 18, 19, and our combined data showed that STAT3 protein and mRNA expression was also negatively correlated with p62 protein and mRNA expression in control group, model group, and α-hederin group (**Fig. 4A, 4B, 5G, 5H, 8F**), similarly with USP5 expression (**Fig. 7D, 8A-E**). These results revealed that USP5 and STAT3 were likely positively correlated. In addition, USP5 may regulate the ubiquitination processes of STAT3 and α-hederin may play a modulatory role in these processes.

### α-hederin diminished the protein stability of STAT3 mediated by USP5

**Figure 9:**
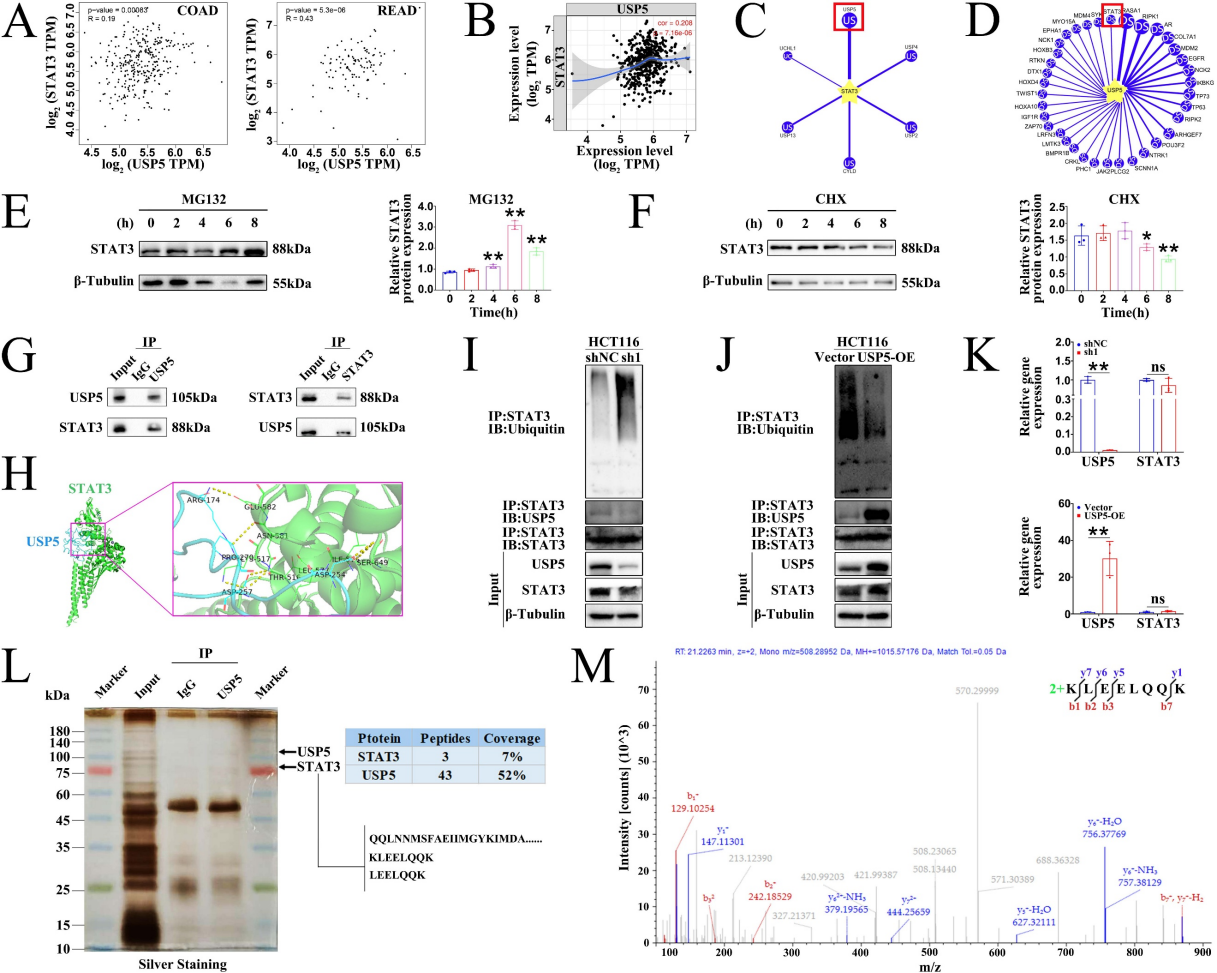
USP5 interacted with STAT3. (A) The correlation between USP5 and STAT3 genes was analysed using the GEPIA database in COAD and READ tissues. (B) The correlation between USP5 and STAT3 genes was analysed using the TIMER database in COAD tissues. (C) Using UbiBrowser 2.0 to predict deubiquitinases that interact with STAT3. The predicted interactors are arranged clockwise in descending order according to the confidence score. (D) Using UbiBrowser 2.0 to predict deubiquitination substrate that interact with USP5. The predicted interactors are arranged clockwise in descending order according to the confidence score. (E, F) Western blotting analysis of STAT3 protein levels in HCT116 cells after treatment with MG132 at 10 µM (E) or CHX at 30 µM (F) for the indicated times, n = 3. (G) The Co-IP experiments were performed to validate the protein interactions between USP5 and STAT3 in HCT116 cells by using USP5 antibody and STAT3 antibody separately. (H) Molecular docking of USP5 and STAT3. (I, J) The lysates of HCT116 cells with stably knockdown of USP5 (I) or overexpressing USP5 (J) were prepared for Co-IP with anti-STAT3 antibody followed by IB analysis of Ubiquitin, USP5 and STAT3 with indicated antibodies. IB analysis of input served as a positive control for all IP/IB assays. (K) Effect of USP5 knockdown or overexpression on mRNA expression levels of STAT3 in HCT116 cells, n = 3. (L) Identification of USP5-interacting proteins using silver staining, and the indicated differential band was cut for LC-MS/MS analysis. (M) The mass spectrum of a unique peptide segment of STAT3. Data are presented as means ± SD. **p < 0.01 versus control group, ns: not significant.

These findings prompted us to investigate the correlation between USP5 and STAT3 expression. Clinical data from the GEPIA (**Fig. 9A**) and TIMER (**Fig. 9B**) databases confirmed that the expression level of USP5 positively correlated with that of STAT3 in COAD and READ tissues. The level of protein expression in the cell depends on the level of transcription and translation of genes as well as on the degradation of proteins. Proteasomes are the primary pathway for the degradation of ubiquitinated proteins. STAT3 is a ubiquitinated protein whose activity is regulated by phosphorylation as well as by ubiquitinated degradation. There are two possible reasons for this decrease in STAT3 protein expression: decreased protein translation and increased protein degradation (or both). STAT3 degradation is dependent on the ubiquitin-proteasome pathway 18, 19. Given that USP5 is a deubiquitinase that can inhibit protein degradation by deubiquitination and that USP28, a homologous protein of USP5, is the only STAT3 DUB reported to date and has been studied in NSCLC 19, we wondered whether USP5 could directly interact with and deubiquitinate STAT3 to inhibit the ubiquitinated degradation of STAT3 and stabilize the STAT3 protein. Notably, the UbiBrowser 2.0 database predicted that USP5 was a potential DUB of STAT3 with the highest confidence score of 0.894 among the six predicted candidate deubiquitinases (**Fig. 9C**). Likewise, the UbiBrowser 2.0 database also predicted that STAT3 was a deubiquitination substrate of USP5 with the high confidence score of 0.894 (**Fig. 9D**).

Next, we determined whether STAT3 undergo ubiquitination in CRC cells. Hence, we treated HCT116 cells with proteasome inhibitor MG132 and protein synthesis inhibitor cycloheximide (CHX). The results showed that STAT3 protein levels increased after treatment with MG132 (**Fig. 9E**) but decreased after treatment with CHX (**Fig. 9F**). These results revealed that STAT3 was modified by ubiquitination. We further performed Co-IP experiments in HCT116 cells and colon tissues of AOM/DSS-treated mice to analyze whether STAT3 deubiquitination was mediated by USP5. The results showed that the proteins recruited by USP5-beads contained STAT3, and the proteins recruited by STAT3-beads contained USP5 (**Fig. 9G**), confirming the endogenous interaction between USP5 and STAT3 in HCT116 cells. Docking of the protein-protein interface interaction of USP5 and STAT3 was also performed (**Fig. 9H**), which provided additional evidence for the structural basis of the USP5-STAT3 interaction. Furthermore, gain- and loss-of-function experiments in HCT116 cells showed that USP5 knockdown accelerated STAT3 ubiquitination and decreased STAT3 protein expression (**Fig. 9I**), whereas USP5 overexpression reduced STAT3 ubiquitination and increased the STAT3 protein level (**Fig. 9J**). Meanwhile, knockdown or overexpression of USP5 had no effects on STAT3 mRNA levels (**Fig. 9K**). Based on the aforementioned findings, we further revealed the interaction between USP5 and STAT3 through silver staining and IP-coupled LC-MS/MS analysis (**Fig. 9L**), and the mass spectrum of a unique peptide segment of STAT3 was shown in** Fig. 9M**. Collectively, these findings indicated that USP5 stabilizes STAT3 by directly binding to STAT3 and decreasing its ubiquitination level, thereby promoting colorectal tumorigenesis.

**Figure 10:**
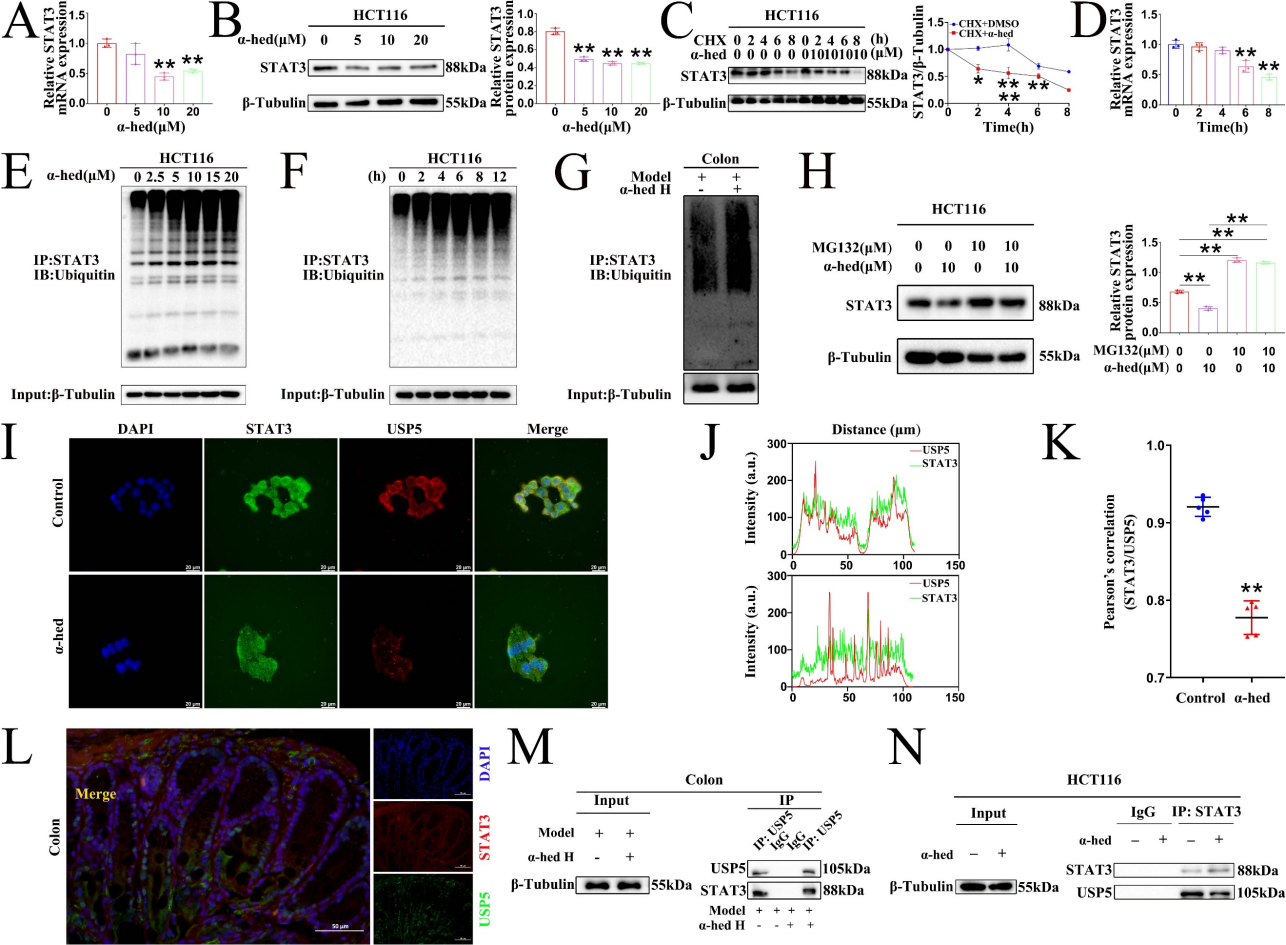
α-hederin diminished the protein stability of STAT3 mediated by USP5. (A, B) Using qPCR and western blotting methods to assess STAT3 mRNA (A) and protein (B) levels in HCT116 cells treated with various concentrations of α-hederin for 24 h, n = 3. (C) IB assay was performed to detect STAT3 expression in HCT116 cells treated with either DMSO or 10 µM α-hederin alongside 30 µM CHX at the indicated times, n = 3. (D) The STAT3 mRNA level in HCT116 cells after treatment with 10 µM α-hederin for the indicated times, n = 3. (E, F) α-hederin-induced ubiquitination of STAT3 was analyzed by Co-IP with an anti-STAT3 antibody followed by western blotting with antibodies against the Ubiquitin protein after treatment with different concentrations of α-hederin (0, 2.5, 5, 10, 15, and 20 μmol·L^-1^) (E) or different times (0, 2, 4, 6, 8, and 12 h) (F) in HCT116 cells. (G) The ubiquitination of STAT3 was analyzed in colon tissues of model and α-hederin H groups of CRC-mice by Co-IP. (H) The protein expression of STAT3 in HCT116 cells treated with a combination of α-hederin (10 µM) and the proteasome inhibitor MG132 (10 µM) using IB assay, n = 3. (I) Co-localization of USP5 with STAT3 in HCT116 cells treated with or without α-hederin using confocal observation, and the qualitative (J) and quantitative (K) analysis of co-localization were performed with Image J, n = 5. Scale bar = 20 μm. (L) Co-localization of USP5 with STAT3 in colon tissue of mice in control group. Scale bar = 50 μm. (M, N) The Co-IP experiments were performed to validate α-hederin inhibition of USP5 binding to STAT3 in colon tissues of CRC-mice (M) and HCT116 cells (N).

To comprehensively investigate how α-hederin decreased STAT3 expression at the molecular level, we further analyzed the levels of STAT3 at the transcriptional and translational levels in HCT116 cells treated with α-hederin. Our findings showed that consistent with *in vivo* result (**Fig. 4B**), α-hederin treatment also decreased the mRNA level of STAT3 in HCT116 cells (**Fig. 10A**), suggesting that α-hederin may downregulate STAT3 expression at the transcriptional level. Additionally, consistent with *in vivo* result, α-hederin treatment also decreased the protein level of STAT3 in HCT116 cells (**Fig. 10B**). Interestingly, when α-hederin was combined with CHX, STAT3 had a shorter half-life in the α-hederin-treated groups than in the DMSO groups (**Fig. 10C**), indicating that α-hederin may also modulate STAT3 by distinctly diminishing its stability at the post-translation level. After incubation of CHX with α-hederin for 2 h, STAT3 protein expression in HCT116 cells was significantly reduced (**Fig. 10C**), whereas α-hederin had no significant effect on STAT3 mRNA expression levels even after 2 or 4 h of incubation with CHX (**Fig. 10D**). Therefore, it was hypothesized that α-hederin mainly decreased STAT3 expression by degrading STAT3 at the post-translational level. So, we focused on the post-translational modification mechanism of α-hederin involved in STAT3 degradation in CRC progression. We further analyzed the ubiquitination of STAT3 protein after α-hederin treatment by protein ubiquitination assay. Encouragingly, endogenous Co-IP results showed that α-hederin treatment significantly increased the ubiquitination of STAT3, and in a dose- (**Fig. 10E**) and time-dependent manner (**Fig. 10F**), making it susceptible to proteasomal digestion. A significant increase in STAT3 ubiquitination was also observed in CRC tissues after α-hederin treatment (**Fig. 10G**) in parallel, and western blot experiments showed that the proteasome inhibitor MG132 reversed the degradation of STAT3 by α-hederin (**Fig. 10H**). These results consistently confirmed the involvement of the ubiquitin-proteasome pathway in the degradation of STAT3 by α-hederin. **Fig. 4B** showed that the synthesis rate of STAT3 decreased after α-hederin treatment. In addition, **Fig. 4A** showed that the STAT3 phosphorylation level was reduced, indicating a decrease in the functional activity of STAT3 after α-hederin treatment, combined with the significant increase in STAT3 ubiquitination after α-hederin treatment, ultimately resulting in a significant decrease in STAT3 expression.

As α-hederin was crucial for STAT3 ubiquitination and directly targeted USP5, elucidating the mechanisms was imperative. Confocal microscopy results revealed that USP5 co-localized with STAT3 in HCT116 cells, however, α-hederin weakened the interaction between USP5 and STAT3 (**Fig. 10I**), and the co-localization qualitative (**Fig. 10J**) and quantitative (**Fig. 10K**) analysis using ImageJ confirmed the weak spatial co-expression of USP5 with STAT3 after α-hederin treatment. Likewise, USP5 also interacted with STAT3 in the colon tissues of mice in control group (**Fig. 10L**), however, compared with model group, the colon tissues of mice in α-hederin H group exhibited the weak mutual binding of STAT3 and USP5 (**Fig. 10M**). Correspondingly, the Co-IP results in HCT116 cells also verified the interaction between STAT3 and USP5, but α-hederin attenuated the reciprocal interaction between these two proteins (Fig. 10N), which in turn disrupted the protein stability of STAT3 mediated by USP5.

Proteins tagged with ubiquitin are recognized and degraded by the proteasome, and DUBs are responsible for removing the ubiquitin tag and protecting the protein from degradation, thereby stabilizing the target protein. When α-hederin inhibited the expression of USP5, the DUB of STAT3, the protein stability of STAT3 was diminished and its expression level decreased. These results showed that USP5 was significantly upregulated during CRC progression and played a critical role in stabilizing STAT3 protein, whereas α-hederin impeded the development of CRC by directly targeting USP5, an oncoprotein, and inhibited USP5 expression as well as weakened its interaction with STAT3, thereby disrupting STAT3 protein stability mediated by USP5 and increased the level of STAT3 ubiquitination. Our findings address a critical gap in understanding STAT3 deubiquitination in CRC by identifying USP5 as a novel DUB for STAT3 and demonstrating its oncogenic role in CRC progression, and highlight the potential of the USP5/STAT3 axis as a therapeutic target for CRC for the first time, indicating that strategically promoting STAT3 degradation by inhibiting USP5 may be a promising approach for CRC prevention and treatment. Our study provided compelling evidences that the natural compound α-hederin exerts anti-tumor effects by directly targeting USP5, disrupting its interaction with STAT3, and inhibiting STAT3 deubiquitination-a previously unreported mechanism.

## Discussion

Although tremendous efforts have been made to develop effective treatments for CRC, late-stage cancer is difficult to treat, and survival rates are low 1. Therefore, interventions during the early stages of CRC progression are urgently needed. It is well known that chronic inflammation is a high-risk factor for inducing CRC 2, and patients with inflammatory bowel disease are at a high risk of developing CRC 34. Therefore, there is an urgent need to develop effective and safe drugs to halt CRC progression by reducing chronic inflammation. Colitis-associated CRC is a stepwise process from inflammation to cancer 58. In previous studies, we have demonstrated that α-hederin inhibited the malignant transformation of IECs 12, 13. Here, we further explored the role and underlying mechanism of α-hederin on the transformation of colitis carcinoma in CRC-mice induced by AOM/DSS.

We firstly investigated the anti-inflammatory effects of α-hederin on cellular inflammatory model in NCM460 cells stimulated by LPS. Our results showed that α-hederin treatment could effectively inhibit the inflammatory response induced by LPS *in vitro* (**Fig. 1**). Further studies demonstrated that α-hederin treatment helped to inhibit tumorigenesis and protect the intestinal barrier function (**Fig. 2**) and reduce intestinal inflammation (**Fig. 3**) in colon tissues of CRC-mice induced by AOM/DSS. Given that p38 MAPK and STAT3 pathways are activated by inflammation, and are prominent stress responsive pathways that regulate tumor cell survival and proliferation and have been identified as the important contributor to CRC development 38, we further detected whether α-hederin inhibited AOM/DSS-induced the formation and growth of CRC through p38 MAPK/STAT3 signaling. Our results showed that α-hederin treatment significantly inhibited colorectal tumor formation and growth by inhibiting p38 MAPK/STAT3 inflammatory-cancer transformation pathway and attenuating oxidative stress (**Fig. 4**). Noteworthily, activated p38 MAPK/STAT3 signaling is required for inducing autophagy 46, which enhances the proliferation and survival of tumor cells. It has been reported that the increased expression of p62, an autophagy inhibitor 48, can inhibit inflammation 49, 50, and the decreased expression of Beclin, an autophagy marker, can reduce inflammation 51. We noticed that compared with the control group, p62 was expressed at lower levels, whereas Beclin was highly expressed in the colon tissues of CRC mice, which was consistent with a previous report 52, demonstrating that p38 MAPK/STAT3 signaling was activated, followed by the induction of autophagy in the progression of CRC, which led to a positive feedback loop that amplified inflammation. However, α-hederin reversed these effects in CRC mice (**Fig. 4**).

STAT3 is a key oncogene with dual functions of signal transduction and transcriptional activation, and its activation is typically constitutive and requires phosphorylation at Tyr705, which could be regulated by p62 56. Here, we revealed that p62 interacted with STAT3 *in vitro* and *in vivo*, wheras α-hederin increased the expression of p62 protein, which plays an anti-inflammatory role 49, 50, and decreased p-STAT3 (Tyr705) expression in colon tissues of CRC-mice, as well as weakened the interaction between p62 and STAT3, and reduced the translocation of STAT3 from the cytoplasm to the nucleus. This may result in inhibition of STAT3 phosphorylation at Tyr705 followed by inhibition of STAT3 constitutive activation in colon tissues of CRC-mice (**Fig. 4, 5**), thereby inhibiting the transformation of colitis carcinoma in CRC-mice. These findings further validated the role of p62 in the regulation of STAT3 phosphorylation at Tyr705 56.

Mechanistically, the HuProt^TM^ V4.0 21K human proteome microarray was used to identify the target proteins of α-hederin. We found that these potential positive binding proteins of α-hederin were mainly enriched in the cell cycle pathway that closely related to cell proliferation and involved in negative regulation of stress-activated protein kinase signaling cascade and negative regulation of stress-activated MAPK cascade biological processes (**Fig. 6**), which was consistent with our experimental results in **Fig. 4**. We subsequently validated that α-hederin inhibited proliferation and promoted apoptosis of colorectal tumor cells* in vivo* and *in vitro*. Among the proteins interacting with α-hederin, what concerns us most was USP5 protein, a DUB. Several studies have reported that aberrant DUB function is associated with cancers 21, 22, and targeting DUBs is very attractive for clinical translation 23, 24. However, there are few clues as to how USP5 acts on CRC. In our study, we subsequently confirmed that USP5 as a direct target of α-hederin by molecular docking, CETSA, DARTS, and streptavidin-biotin pull-down assays (**Fig. 6**). We also observed that USP5 was highly expressed in CRC tissues (GEPIA and TIMER databases), in samples from patients with CRC, in colon tissues of CRC-mice, and in CRC cells (**Fig. 7**). This is in line with the findings of the previous study showing that USP5 was highly expressed in primary CRC tissues of patients, and correlated with disease stage and overall survival 27. This reveals that USP5 may be an oncoprotein in CRC, and that α-hederin could decrease USP5 expression either in a cellular inflammation model, CRC mice, or CRC cells (**Fig. 8**).

In a subsequent study, we were delighted to discover that until now the USP28, which is homologous to USP5, is the only known DUB of STAT3 that has been studied in NSCLC 19. USP5 is involved in inflammation and upregulates the expression of inflammatory factors in hepatocyte inflammation 26. We have demonstrated that STAT3 was activated in CRC-mice, forming a positive feedback loop that amplifies intestinal inflammation and maintains the STAT3 signal, but STAT3 activation was attenuated by α-hederin in **Fig. 4**. As the decrease in STAT3 protein expression could be accounted for the decreased protein translation and increased protein degradation (or both), and the degradation of STAT3 protein is proven to be dependent on the ubiquitin-proteasome pathway 18, 19, and the clinical success of bortezomib 59 that targets ubiquitin-proteasome pathway-mediated protein degradation in cancer cells, illustrates the importance of ubiquitin-mediated signaling in cancer; however, the ubiquitination mechanism of STAT3 in CRC is still unclear, which stimulated further research on STAT3 ubiquitination. Considering the above reports, combined with our findings, we speculated that like USP28, USP5 could deubiquitinate STAT3 to inhibit STAT3 ubiquitinated degradation. Surprisingly, clinical data from the GEPIA and TIMER databases confirmed that the expression level of USP5 was positively correlated with that of STAT3 in CRC tissues. USP5 was predicted to be the most promising deubiquitinase of STAT3 and STAT3 was also predicted to be a potential deubiquitination substrate of USP5 in the UbiBrowser 2.0 database. We first confirmed that STAT3 was modified by ubiquitination in HCT116 cells, and then performed Co-IP and molecular docking to confirm the interaction between USP5 and STAT3. Moreover, USP5 knockdown promoted ubiquitination and proteasomal degradation of STAT3, whereas USP5 overexpression stabilized STAT3. Neither the knockdown nor overexpression of USP5 affected STAT3 gene expression. Importantly, the interaction between USP5 and STAT3 was further revealed using LC-MS/MS (**Fig. 9**). These mechanistic studies further demonstrated that USP5 promoted tumorigenesis by interacting with STAT3 and stabilizing its protein levels through deubiquitination.

Next, we delved into the mechanism through which α-hederin regulates STAT3 ubiquitination. Consistent with *in vivo* findings (**Fig. 4**), α-hederin decreased STAT3 expression both at the transcriptional level and at the post-translation level in HCT116 cells, but through in-depth research we found that α-hederin mainly decreased STAT3 expression by degrading STAT3 at the post-translational level, and α-hederin treatment significantly increased the ubiquitination of STAT3 *in vitro* and *in vivo*, which could be reversed by proteasome inhibitor MG132, further confirmed the engagement of the ubiquitin-proteasome pathway in the degradation of STAT3 by α-hederin. In the subsequent studies, we found that α-hederin targeted USP5, decreased its expression level, and dismished its interaction with STAT3 both *in vitro* and *in vivo*. This disruption of USP5-mediated STAT3 stability led to increased STAT3 ubiquitination, reduced STAT3 expression, and ultimately inhibited the progression of colitis-associated cancer transformation (**Fig. 10**).

**Figure 11:**
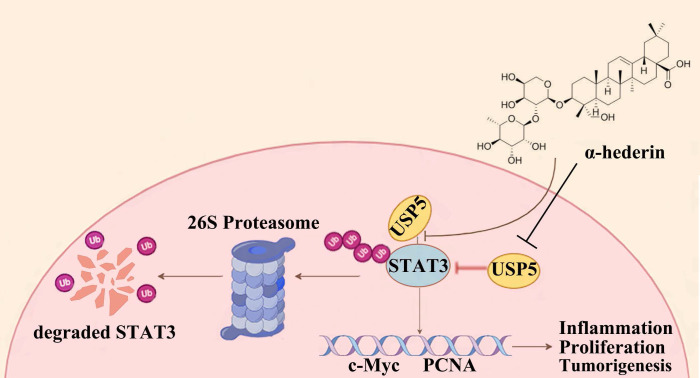
Graphical abstract: The proposed mechanism of α-hederin inhibits colitis-associated colorectal cancer.

A deeper understanding of the upstream regulatory mechanisms of STAT3 is important for discovering novel targets for cancer treatment. In this study, we identified USP5 as a true regulator of STAT3 and revealed a previously uncharacterized role of USP5 in STAT3 degradation. USP5 directly interacts with STAT3 and enhances the stability of STAT3 proteins through deubiquitination, thereby promoting colorectal tumorigenesis. Our findings revealed that α-hederin served as a potential therapeutic candidate for the prevention and treatment of CRC by suppressing STAT3 activation mainly through targeting and decreasing USP5 expression and inhibiting the deubiquitylation of STAT3 mediated by USP5, promoting STAT3 ubiquitin-proteasome degradation, and thereby reducing the inflammatory response and halting the proliferation of malignant IECs, providing a promising therapeutic strategy for CRC prevention and treatment (**Fig. 11**).

## Conclusions

To the best of our knowledge, this is the first report identifying α-hederin as a promising preventive agent for inhibiting colorectal tumorigenesis by directly targeting USP5, reducing its expression and interaction with STAT3, thereby disrupting USP5-mediated STAT3 deubiquitination.

## Supplementary Material

Supplementary figures and tables.

## Figures and Tables

**Table 1:** Clinical information of CRC patients enrolled in the research.

Patient	Gender	Age	Cancer subtype	TNM stage
1	female	53	Colon carcinoma	IV
2	male	63	Rectal carcinoma	IIA
3	female	71	Rectal carcinoma	IIIA
4	female	70	Rectal carcinoma	IIIB
5	male	70	Colon carcinoma	IIA
6	male	63	Colon carcinoma	IIA
7	male	75	Colon carcinoma	I

## Abbreviations

AOM: azoxymethane
CRC: colorectal cancer
CETSA: cellular thermal shift assay
CHX: cycloheximide
DUB: deubiquitinating enzyme
DARTS: drug affinity responsive target stability
DSS: dextran sodium sulfate
DMSO: dimethyl sulfoxide
DAB: 3,3-N-DiaminobenzidineTertrahydrochloride
DHE: dihydroethidium
ELISA: enzyme-linked immunosorbent assay
H&E: hematoxylin and eosin
IECs: intestinal epithelial cells
IF: immunofluorescence
IL-6: interleukin-6
IL-1β: interleukin-1 beta
IL-10: interleukin-10
IHC: immunohistochemistry
IP: immunoprecipitation
LPS: lipopolysaccharide
MAPK: mitogen-activated protein kinase
MTT: 3-(4,5-dimethylthiazol-2-yl)-2,5 diphenyl tetrazolium bromide
NSCLC: non-small-cell lung cancer
NF-κB: nuclear factor-κappa B
PTM: post-translational modifications
PBS: phosphate-buffered saline
PBST: phosphate-buffered saline with Tween 20
PVDF: polyvinylidene difluoride
PFA: paraformaldehyde
PCNA: proliferating cell nuclear antigen
qPCR: quantitative real-time polymerase chain reaction
RT: room temperature
STAT3: signal transducer and activator of transcription 3
SDS-PAGE: sodium dodecyl sulfate-polyacrylamide gel electrophoresis
TUFM: Tu translation elongation factor
TNF-α: tumor necrosis factor-alpha
USP5: ubiquitin specific peptidase 5
ZO-1: zonula occludens-1
