# Supplementary material for: α-hederin Targets USP5 to Inhibit Colorectal Tumorigenesis by Disrupting STAT3 Deubiquitination
Source: Int J Biol Sci. 2025 Oct 20;21(15):6697–722. doi: 10.7150/ijbs.119868 (PMC12631106; doi:10.7150/ijbs.119868)
Supplement: Supplementary file 1 — Supplementary figures and tables. [file ijbsv21p6697s1.pdf]

Supporting Information for
Original article
α-hederin Targets USP5 to Inhibit Colorectal Tumorigenesis by Disrupting STAT3 Deubiquitination

Supplementary Tables

Table S1: Primer sequences for qPCR assay.

Gene Symbol	Species	Forward sequences (5'-3')	Reverse sequences (5'-3')
IL-6	Human	ACTCACCTCTTCAGAACGAATTG	CCATCTTTGGAAGGTTCAGGTTG
IL-1β	Human	TGATGGCTTATTACAGTGGCAA	TAGTGGTGGTCGGAGATTCG
TNF-α	Human	TGCACTTTGGAGTGATCGGC	ACTCGGGGTTCGAGAAGATG
USP5	Human	ACCAGAAAGTGTGTGCCTCC	CCCCTGCCCTCATTGGTAAG
p62	Human	TTGCGCCCATTTAGAGGATGT	GGCCCTGGCATTGTTCTTACA
STAT3	Human	ACCAGCAGTATAGCCGCTTC	GCCACAATCCGGGCAATCT
β–Actin	Human	GAGCACAGAGCCTCGCCTTT	TCATCATCCATGGTGAGCTGG
IL-6	Mouse	CCAAGAGGTGAGTGCTTCCC	CTGTTGTTCAGACTCTCTCCCT
IL-1β	Mouse	AGCATCCAGCTTCAAATC	CTTCTCCACAGCCACAAT
TNF-α	Mouse	CAGGCGGTGCCTATGTCTC	CGATCACCCCGAAGTTCAGTAG
IL-10	Mouse	CTTACTGACTGGCATGAGGATCA	GCAGCTCTAGGAGCATGTGG
ZO-1	Mouse	GCCGCTAAGAGCACAGCAA	TCCCCACTCTGAAAATGAGGA
Occludin	Mouse	TTGAAAGTCCACCTCCTTACAGA	CCGGATAAAAAGAGTACGCTGG
STAT3	Mouse	AGAACCTCCAGGACGACTTTG	TCACAATGCTTCTCCGCATCT
c-Myc	Mouse	ATGCCCCTCAACGTGAACTTC	CGCAACATAGGATGGAGAGCA
PCNA	Mouse	TTTGAGGCACGCCTGATCC	GGAGACGTGAGACGAGTCCAT
β-Catenin	Mouse	CCCAGTCCTTCACGCAAGAG	CATCTAGCGTCTCAGGGAACA
USP5	Mouse	GTGTTACCGACGATCCGTGTC	GTTCATGCAGATATAGAGGCCAC
p62	Mouse	GAGTCCCTCTCCCAGATGCT	GCCAAGACACTGGGCCTATC
β–Actin	Mouse	GATCAGCAAGCAGGAGTACGA	GGGTGTAAAACGCAGCTCA

Table S2: Primer sequences of pLVshUSP5.

Gene Symbol	Species	Sequences (5'-3')
shRNA	Human	GATCCACTACCGTTGTTATAGGTGTTCAAGAGACACCTATAACAACGGTAGTTTTTT
shUSP5#1	Human	gatcgGACCACACGATTTGCCTCATTCTCGAGAATGAGGCAAATCGTGTGGTCTTTTTT
shUSP5#2	Human	gatcgGATAGACATGAACCAGCGGATCTCGAGATCCGCTGGTTCATGTCTATCTTTTTT
shUSP5#3	Human	gatcgACAGTATGTGGAGAGACATTTCTCGAGAAATGTCTCTCCACATACTGTTTTTTT

Table S3: Primer sequences of USP5-OE.

Gene Symbol	Species	Sequences (5'-3')
USP5-OE	Human	GCCACCATGGCGGAGCTGAGTGAGGAGGCGCTGCTGTCAGTATTACCGACGATCCGG<br>GTCCCTAAGGCTGGAGACCGGGTCCACAAAGACGAGTGCGCCTTCTCCTTCGACACG<br>CCGGAGTCTGAGGGGGGCCTCTACATCTGTATGAACACGTTTCTGGGCTTTGGGAAA<br>CAGTATGTGGAGAGACATTTCAATAAGACCGGCCAGCGAGTCTACTTGCACCTCCGG<br>CGGACCCGGCGCCCGAAAGAGGAGGACCCTGCTACAGGCACTGGAGACCCACCCCG<br>GAAGAAGCCCACGCGGCTGGCTATTGGTGTTGAAGGCGGATTTGACCTTAGCGAGGA<br>GAAGTTTGAATTAGACGAGGATGTGAAGATTGTCATTTTGCCAGATTACCTGGAGAT<br>TGCCCGGGATGGACTGGGGGGACTGCCTGACATTGTCAGAGATCGGGTGACCAGTGC<br>AGTGGAGGCCCTACTGTCGGCCGACTCAGCCTCCCGCAAGCAGGAGGTGCAGGCATG<br>GGATGGGGAAGTACGGCAGGTGTCTAAGCATGCCTTCAGCCTCAAGCAGTTGGACAA<br>CCCTGCTCGAATCCCTCCCTGTGGCTGGAAGTGCTCCAAGTGTGACATGAGAGAGAA<br>CCTGTGGCTCAACCTGACTGATGGCTCCATCCTCTGTGGGCGACGCTACTTCGATGGC

AGTGGGGGCAACAACCACGCTGTGGAGCACTACCGAGAGACAGGCTACCCGTTAGCT
GTCAAGCTGGGCACCATCACCCCTGATGGAGCTGACGTGTACTCATATGATGAGGAT
GACATGGTCCTGGACCCCAGCCTGGCTGAGCACCTGTCCCACTTCGGCATCGACATG
CTGAAGATGCAGAAGACAGACAAGACGATGACTGAGTTGGAGATAGACATGAACCA
GCGGATTGGTGAATGGGAGCTGATCCAGGAGTCAGGTGTGCCACTCAAGCCCCTGTT
TGGGCCTGGCTACACAGGCATCCGGAACCTGGGTAACAGCTGCTACCTCAACTCTGT
GGTCCAGGTGCTCTTCAGCATCCCTGACTTCCAGAGGAAGTATGTGGATAAGCTGGA
GAAGATCTTCCAGAATGCCCCGACGGACCCTACCCAGGATTTCAGCACCCAGGTGGC
CAAGCTGGGCCATGGCCTTCTCTCCGGGGAGTATTCCAAGCCAGTACCGGAGTCGGG
CGATGGGGAGCGGGTGCCAGAACAGAAGGAAGTTCAAGATGGCATTGCCCCTCGGA
TGTTCAAGGCCCTCATCGGCAAGGGCCACCCTGAATTCTCCACCAACCGGCAGCAGG
ATGCCCAGGAGTTCTTCCTTCACCTTATCAACATGGTGGAGAGGAATTGCCGGAGCTC
TGAAAATCCTAATGAAGTGTTCCGCTTCTTGGTGGAGGAAAAGATCAAGTGCCTGGC
CACAGAGAAGGTGAAGTACACCCAGCGAGTTGACTACATCATGCAGCTGCCTGTGCC
CATGGATGCAGCCCTTAACAAAGAGGAGCTTCTGGAGTACGAGGAGAAGAAGCGGC
AAGCCGAAGAGGAGAAGATGGCACTGCCAGAACTGGTTCGGGCCCAGGTGCCCTTC
AGCTCTTGCCTGGAGGCCTACGGGGCCCCTGAGCAGGTCGATGACTTCTGGAGCACG
GCCCTGCAGGCCAAGTCAGTAGCTGTCAAGACCACACGATTTGCCTCATTCCCTGACT
ACCTGGTCATCCAGATCAAGAAGTTCACCTTCGGCTTAGACTGGGTGCCCAAGAAAC
TGGATGTGTCCATCGAGATGCCAGAGGAGCTCGACATCTCCCAGTTGAGGGGCACAG
GGCTGCAGCCCGGAGAGGAGGAGCTGCCAGACATTGCCCCACCCCTGGTCACTCCGG
ATGAGCCCAAAGGTAGCCTTGGTTTCTATGGCAACGAAGACGAAGACTCCTTCTGCT
CCCCTCACTTCTCCTCTCCGACATCGCCCATGCTGGATGAATCAGTCATCATCCAGCT
GGTGGAGATGGGATTCCCTATGGACGCCTGCCGCAAAGCTGTCTACTACACGGGCAA
CAGCGGGGCTGAGGCCGCCATGAACTGGGTCATGTCACACATGGATGATCCAGATTT
TGCAAACCCCCTCATCCTGCCTGGCTCTAGTGGGCCGGGCTCCACAAGCGCAGCAGC
CGACCCCCCTCCTGAGGACTGTGTGACCACCATTGTCTCCATGGGCTTCTCCCGGGAC
CAGGCCTTGAAAGCGCTGCGGGCCACGAACAATAGTTTAGAACGGGCTGTGGACTGG
ATCTTCAGTCACATTGACGACCTGGATGCTGAAGCTGCCATGGACATCTCAGAGGGC
CGCTCAGCTGCCGACTCCATCTCTGAGTCTGTGCCAGTGGGACCTAAAGTCCGGGAT
GGTCCTGGAAAGTATCAGCTCTTTGCCTTCATTAGTCACATGGGCACCTCTACCATGT
GTGGTCACTACGTCTGCCACATCAAGAAAGAAGGCAGATGGGTGATCTACAATGACC
AGAAAGTGTGTGCCTCCGAGAAGCCGCCCAAGGACCTGGGCTACATCTACTTCTACC
AGAGAGTGGCCAGCTAA

Supplementary Figures

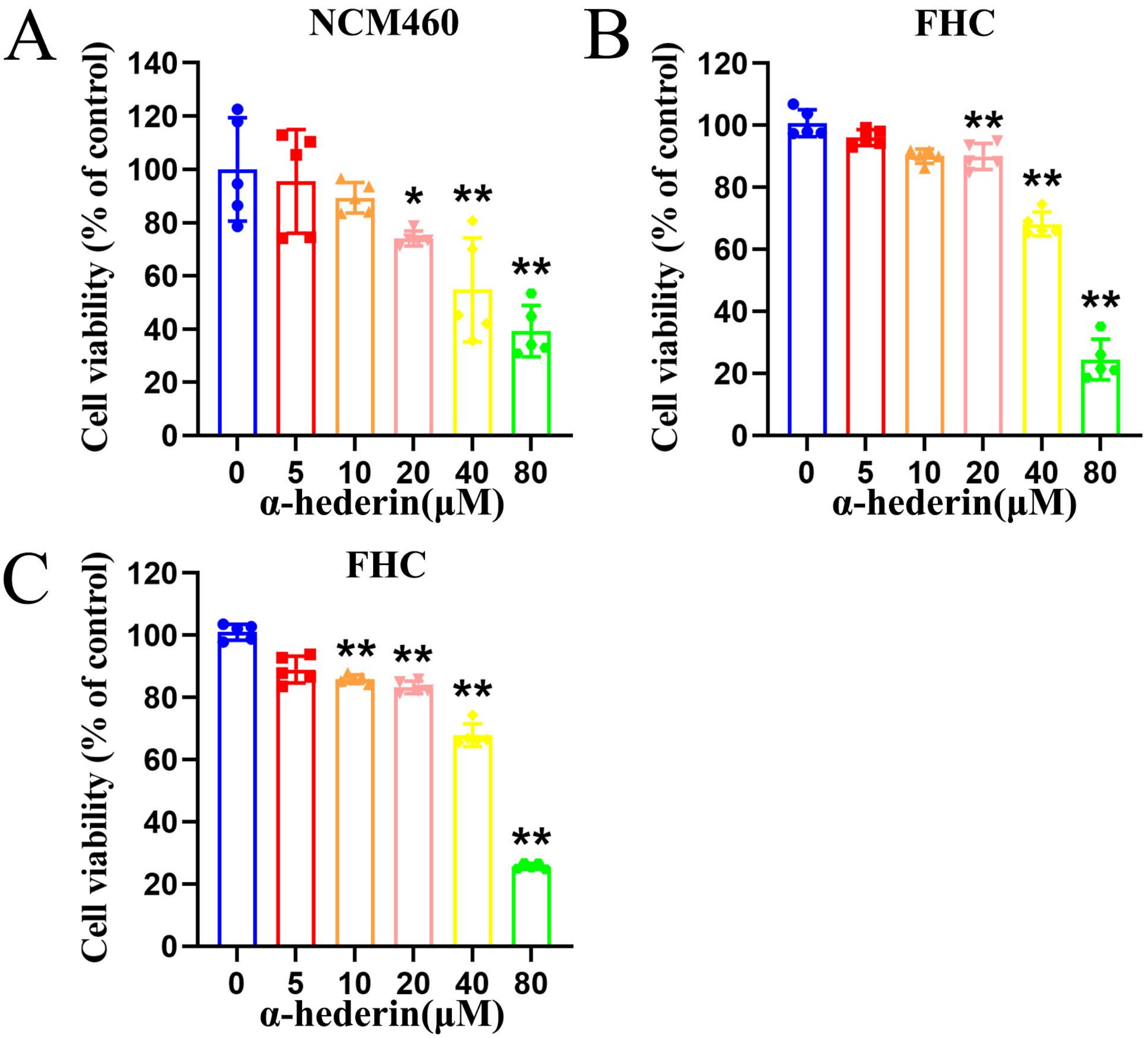

Fig. S1 Cell viability of two different normal human colonic epithelial cell lines NCM460 and FHC treated with different concentrations of α-hederin for 24 and 48 h. (A) Cell viability of NCM460 treated with α-hederin for 48 h at the indicated concentrations, n = 5. (B) Cell viability of FHC treated with α-hederin for 24 h at the indicated concentrations, n = 5. (C) Cell viability of FHC treated with α-hederin for 48 h at the indicated concentrations, n = 5. Data are presented as means ± SD. *$p < 0.05$, **$p < 0.01$ versus control group.

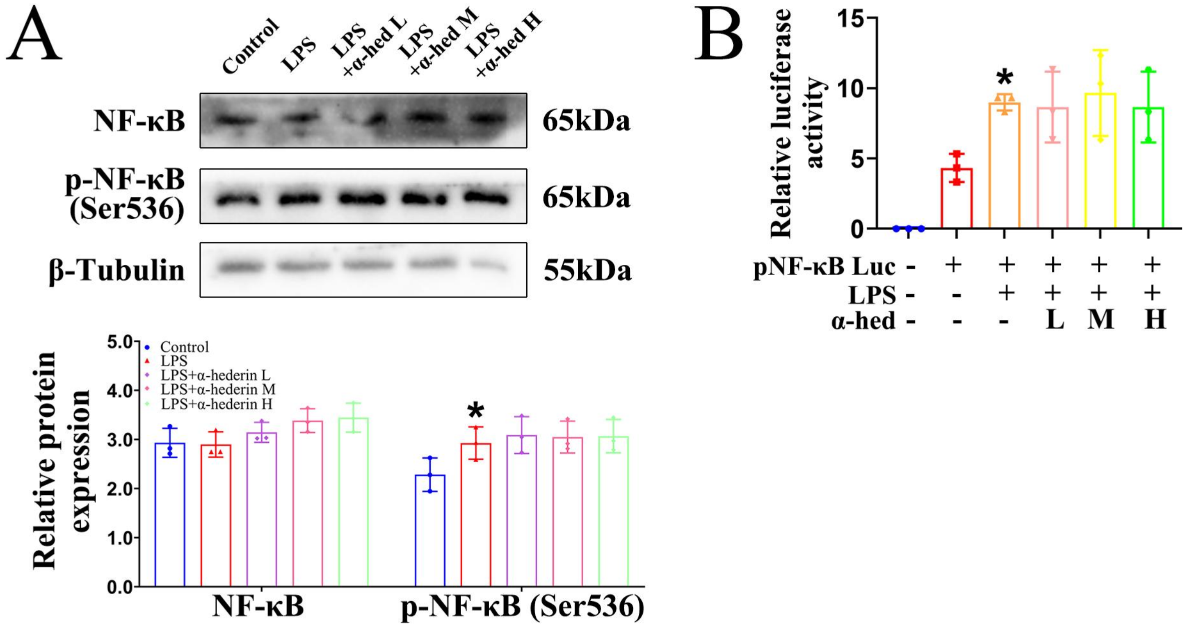

Fig. S2 α-hederin specifically inhibited STAT3 activity. (A) The expression of NF-κB and the levels of phosphorylated NF-κB were detected by western blotting, n = 3. (B) The transcriptional activity of NF-κB was detected by luciferase assay, n = 3. Data are presented as means ± SD. *$p < 0.05$ versus control group.

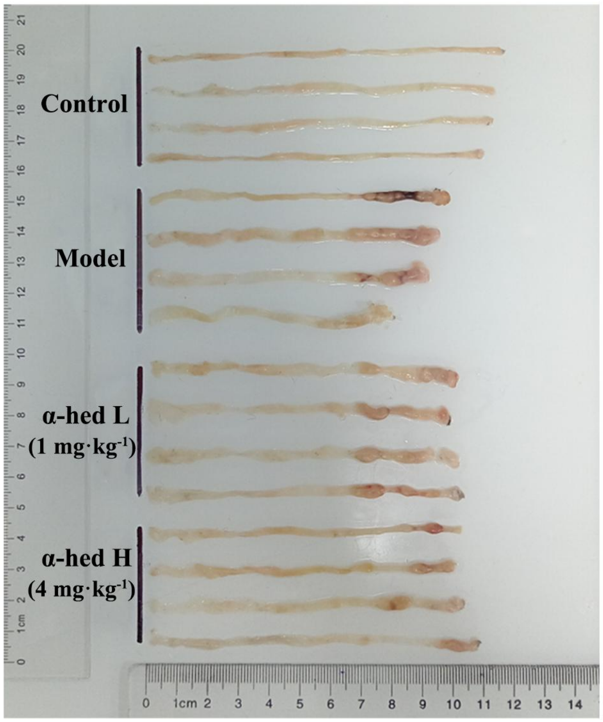

Fig. S3 The gross pathology of the mouse colon.

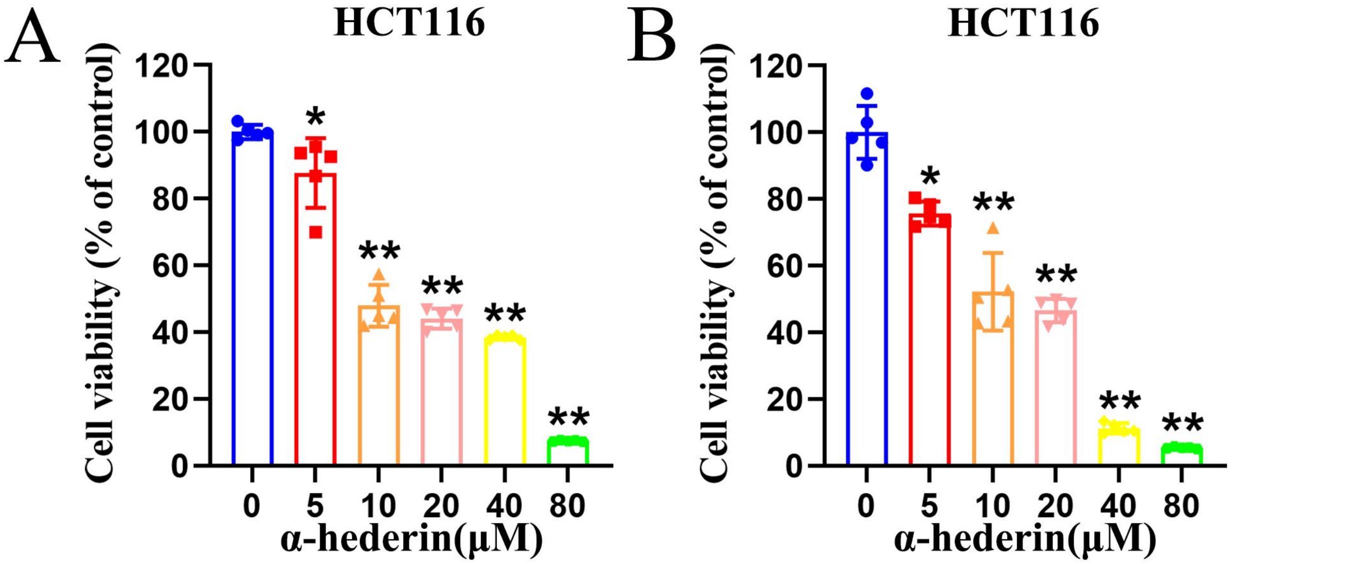

Fig. S4 Cell viability of human CRC cell HCT116 treated with α-hederin for 24 and 48 h at the indicated concentrations. Data are presented as means ± SD. *$p < 0.05$, **$p < 0.01$ versus control group.

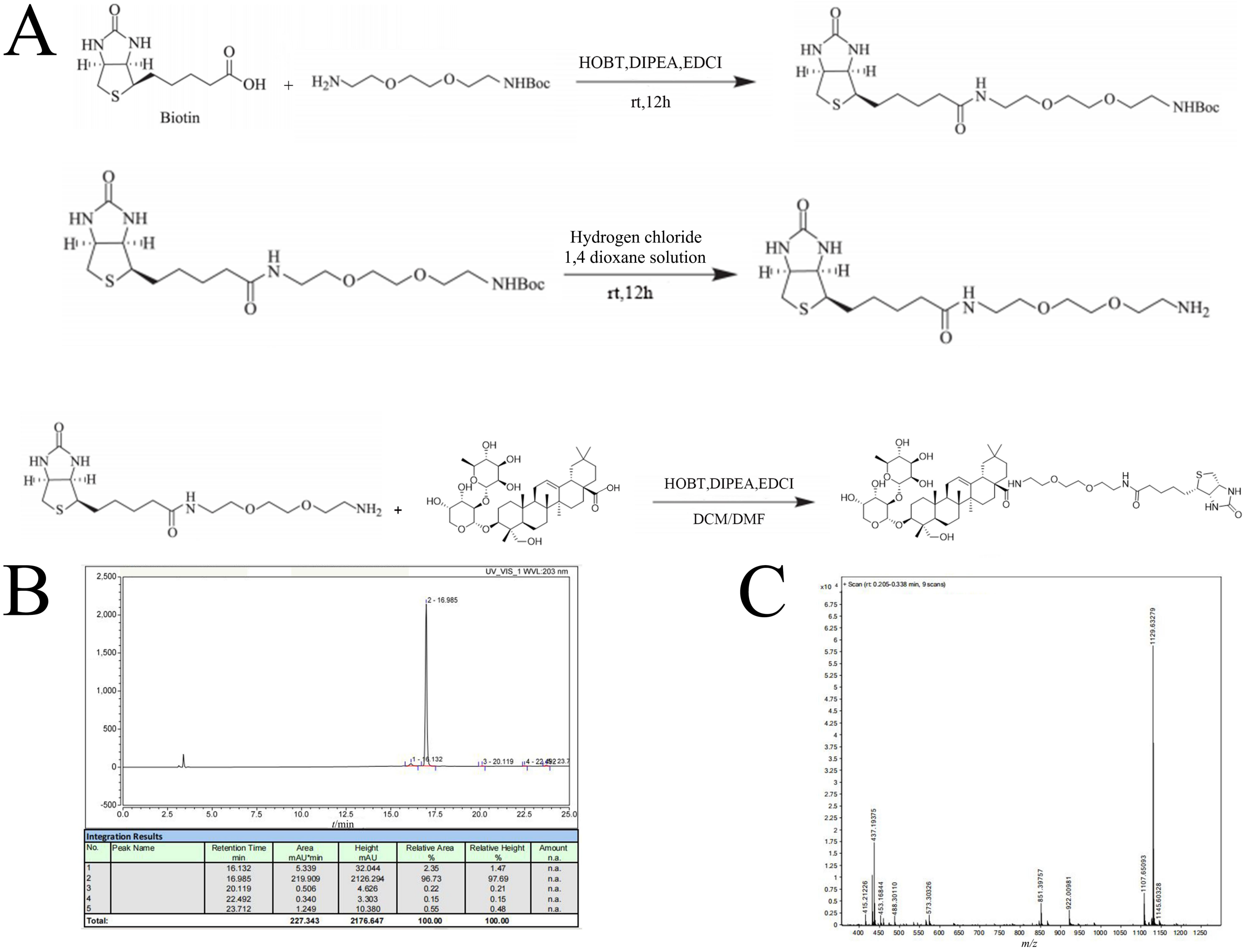

Integration Results							
No.	Peak Name	Retention Time min	Area mAU*min	Height mAU	Relative Area %	Relative Height %	Amount n.a.
1		16.132	5.339	32.044	2.35	1.47	n.a.
2		16.985	219.909	2126.294	96.73	97.69	n.a.
3		20.119	0.506	4.626	0.22	0.21	n.a.
4		22.492	0.340	3.303	0.15	0.15	n.a.
5		23.712	1.249	10.380	0.55	0.48	n.a.
Total:			227.343	2176.647	100.00	100.00	

Fig. S5 The synthesis and purification of Bio-α-hederin. (A) The synthetic route of Bio-α-hederin. (B) The HPLC chromatogram of Bio-α-hederin. (C) High-resolution mass spectra of Bio-α-hederin in positive ion mode.
